# Refinement of Light-Responsive Transcript Lists Using Rice Oligonucleotide Arrays: Evaluation of Gene-Redundancy

**DOI:** 10.1371/journal.pone.0003337

**Published:** 2008-10-06

**Authors:** Ki-Hong Jung, Christopher Dardick, Laura E. Bartley, Peijian Cao, Jirapa Phetsom, Patrick Canlas, Young-Su Seo, Michael Shultz, Shu Ouyang, Qiaoping Yuan, Bryan C. Frank, Eugene Ly, Li Zheng, Yi Jia, An-Ping Hsia, Kyungsook An, Hui-Hsien Chou, David Rocke, Geun Cheol Lee, Patrick S. Schnable, Gynheung An, C. Robin Buell, Pamela C. Ronald

**Affiliations:** 1 Department of Plant Pathology, University of California Davis, Davis, California, United States of America; 2 Appalachian Fruit Research Station, USDA-ARS, Kearneysville, West Virginia, United States of America; 3 J. Craig Venter Institute, Rockville, Maryland, United States of America; 4 Center for Plant Genomics, Iowa State University, Ames, Iowa, United States of America; 5 Functional Genomic Center, Pohang University of Science and Technology, Pohang, Republic of Korea; 6 College of Business Administration, Konkuk University, Gwangjin-gu, Seoul, Korea; Umeå Plant Science Centre, Sweden

## Abstract

Studies of gene function are often hampered by gene-redundancy, especially in organisms with large genomes such as rice (*Oryza sativa*). We present an approach for using transcriptomics data to focus functional studies and address redundancy. To this end, we have constructed and validated an inexpensive and publicly available rice oligonucleotide near-whole genome array, called the rice NSF45K array. We generated expression profiles for light- vs. dark-grown rice leaf tissue and validated the biological significance of the data by analyzing sources of variation and confirming expression trends with reverse transcription polymerase chain reaction. We examined trends in the data by evaluating enrichment of gene ontology terms at multiple false discovery rate thresholds. To compare data generated with the NSF45K array with published results, we developed publicly available, web-based tools (www.ricearray.org). The Oligo and EST Anatomy Viewer enables visualization of EST-based expression profiling data for all genes on the array. The Rice Multi-platform Microarray Search Tool facilitates comparison of gene expression profiles across multiple rice microarray platforms. Finally, we incorporated gene expression and biochemical pathway data to reduce the number of candidate gene products putatively participating in the eight steps of the photorespiration pathway from 52 to 10, based on expression levels of putatively functionally redundant genes. We confirmed the efficacy of this method to cope with redundancy by correctly predicting participation in photorespiration of a gene with five paralogs. Applying these methods will accelerate rice functional genomics.

## Introduction

Large genomes often contain many paralogous genes that are closely related by sequence [1], [2]. Eighty-three percent of the 25,193 predicted human proteins contain regions that significantly match other human proteins [3]. This genomic property is even more pronounced in plant species. For *Arabidopsis*, over 26,500 gene loci have been predicted, and rice and poplar contain more than 40,000 genes [4]. The numbers of genes in sequenced plant species are greater by 2-fold or more than the predicted number of genes for the ancestral angiosperm (12,000–14,000) [4]. This phenomenon can be explained by significant gene duplication events [4]. The Osa1 Version 5 rice genome annotation (formerly known as the TIGR v. 5 annotation, http://rice.plantbiology.msu.edu/) recently identified 3842 rice paralogous gene families consisting of 20,729 protein sequences. Such paralogous gene families are a main source of functional redundancy in mouse and yeast [5]–[7]. This poses a particular obstacle to functional studies since it is often the case that a gene, when mutated, will display no detectable phenotype [5], [8]. Thus, a major challenge facing scientists is identifying which paralog(s) functions in the biological process of interest. Whole genome transcriptomics data are one important tool for informing hypotheses regarding gene function, both for unique and redundant genes [9], [10]. With the goal of advancing functional genomics studies of rice, we have developed and validated an inexpensive, publicly available rice whole genome oligonucleotide (oligo) array.

Monocotyledenous crops in the family *Poaceae*, including rice, wheat, maize, oat and sorghum, are the most important food and feed crops for humans and domesticated animals [11]–[14]. Rice, because of its small genome size, extensive genetic map, available genome sequence and gene expression profiles, and relative ease of transformation has emerged as a model monocot [15]–[21]. While a great deal can be learned through comparison with more distantly related species, a complete understanding of the biology of economically important grasses, including cereals and bioenergy crops, will depend upon a full characterization of the gene complement of a model monocot

Researchers have sequenced the genomes of two distinct subspecies of cultivated rice. The Beijing Genomics Institute (BGI) generated a draft sequence of *O. sativa* subsp. *indica* cultivar 93-11 through shotgun sequencing [22]. In addition, the International Rice Genome Sequencing Project (IRGSP), a consortium of public laboratories, sequenced the genome of *O. sativa* subsp. *japonica* cultivar Nipponbare using a bacterial artificial chromosome (BAC)-by-BAC approach [19]. A well-annotated, publicly available rice genome sequence has stimulated the development of a number of functional genomics resources. One such resource is gene-indexed mutants covering more than half of rice genome [23]. These materials are valuable for identifying gene function either through forward or reverse genetics, in which hypotheses regarding the function of specific genes are tested in corresponding mutants. Another way to gain clues about gene function is to monitor gene expression in response to stimuli or throughout development. The advent of nucleic acid microarrays now makes this possible on a genome scale. Microarray experiments permit biologists to concurrently measure expression levels of thousands of genes in a single experiment through the hybridization of nucleic acid to pre-designed oligos. As listed below, three whole-genome oligo microarray platforms have been developed for rice based on early rice gene predictions (called pseudomolecules) from The Institute for Genomic Research (TIGR) and/or rice full-length cDNAs available from the Knowledge-based Oryza Molecular biological Encyclopedia (KOME, http://cdna01.dna.affrc.go.jp/cDNA/) [23].

1. Yale University and BGI designed an *O. sativa* genome oligo set (Version 1.0) that contains 60,727, 70-mer oligos representing both the *indica* and *japonica* genomes [24], [25]. Oligos were designed from cDNAs, expressed sequence tag (EST) sequences, putative genes based on the BGI rice genome build and other public resources (http://www.operon.com/arrays/oligosets_rice.php).
2. Affymetrix (http://www.affymetrix.com/products/arrays/specific/rice.affx) has developed a rice GeneChip that contains oligos based on approximately 48,564 *japonica* transcripts and 1260 *indica* transcripts. Sequence information used to develop this array includes public content from UniGene Build #52 (May 7, 2004), GenBank® mRNAs (July 13, 2004), and 59,712 putative genes based on TIGR's rice genome annotation release 2.0 [16], [22]. Including control spots, 55,515 probe sets were synthesized and included on this chip. Each set is comprised of 11 probes of 25 nucleotides each [26].
3. Agilent (http://www.chem.agilent.com/scripts/pds.asp?lPage=12133) has released a 44K element oligo array based on rice full-length cDNAs (http://cdna01.dna.affrc.go.jp/cDNA) [27].

To date, most microarray studies in rice have not focused on discovery of gene function *per se*, but instead have provided a profile of a particular organ, environmental response, or genetic background [25], [26], [28], [29]. For example, researchers recently reported the use of the Yale/BGI rice array and a similar array for *Arabidopsis thaliana* to compare and contrast expression profile changes of different organs in rice and Arabidopsis [25] and during light-regulated seedling development [28]. They conclude that light-regulated transcription is more similar between the two species than dark-regulated transcription [28] and that expression of biochemical pathways and protein synthesis genes are more highly correlated than that of transcription factors [25], [28]. Walia et al. [26] reported one of the first uses of the rice Affymetrix array and described profiling of rice responses to salt stress of a tolerant recombinant inbred line and its sensitive parental line. These researchers noted that some of the induced genes fell into physical clusters on the rice chromosomes, including a region associated with a salt-tolerance quantitative trait locus (QTL). Shimono et al. [27] report one of the first uses of the Agilent 44K rice array and one of the first instances of using microarray data for gene function discovery in rice. This study led to the identification of a positive role for a transcription factor gene product, OsWRKY45, in rice defense against a fungal pathogen. However, the basis for further testing of this gene among the ∼300 genes induced under the treatment conditions was based on previous knowledge about the roles of WRKYs in defense responses, and three other related genes examined based on the same criteria yielded no phenotype.

Here, we report construction and validation of a 43,311 oligo rice gene array based on 45,116 gene models from the 61,420 total target sequences present in TIGR rice annotation release 3 [30]. Because this array was supported by the National Science Foundation and is based on 45,116 gene models, it is called the NSF45K array. To validate the functional utility of the NSF45K array, we conducted experiments to identify candidate genes involved in light responses. We hybridized RNAs from four rice varieties exposed to light and dark treatments to the array. With the data resulting from these experiments, we employed five methods to verify the usefulness of the NSF45K array (**Table 1**), including analyzing the sources of variation, GO-term enrichment in lists of light- and dark-induced genes, and comparing the data with rice EST and other microarray data. We then assessed functional redundancy with an approach for integrating expression data with pathway information by analyzing available gene expression profiles from multiple array platforms. For the project, we developed publicly available web-based tools for analysis of gene expression based on rice ESTs and data from other array platforms. These methods and tools will allow users to more accurately refine their candidate gene lists to improve the efficiency of functional testing, greatly accelerating rice gene discovery.

**Table 1:** Strategy employed for validating the data from the rice NSF45K array.

Validation method	Validated parameter	Result
1. Analysis of variation (ANOVA) test	Treatment, slide, samples, other errors.	Treatment is major source of variation (**Figure 1**).
2. Independent experimental analysis	Validation of gene expression patterns using RT-PCR	Expression patterns of 30 highly light-induced genes were confirmed (**Figure S1**).
3. PlantGOSlim analysis	Enrichment analysis of GO terms at four FDRs.	Photosynthesis in biological processes, chloroplast in cellular components, and chlorophyll binding activity in molecular functions were the most meaningful (**Figure 2** **–** **3**, **Figure S4**).
4. Comparison with public transcriptomics data	a. Digital northern of numbers of ESTs in leaf tissue.	Significantly differentially expressed genes are more likely to have leaf-derived EST support than EST-support from other tissues (**Figure 4A** and **4B**).
	b. Comparison with other microarray data using different platform with similar experiments (various intensities of light *vs.* dark).	485 of 887 candidate light induced genes had similar gene expression patterns among different platforms (**Figure 5**).
5. Analysis of a biochemical pathway with the microarray data	Expression of eight components in the photorespiration pathway.	One or more paralogous genes from all 8 steps of the pathway showed significant induction in the light (**Figure 6B**).
		Two steps in the pathway showed light induction from more than one paralog^a^, suggesting functional redundancy (**Figure 6B** and **6C**).

a Paralogs are genes related by duplication within a genome and as a wider meaning, indicate gene families likely to have functional redundancy.

## Results and Discussion

### Light *vs.* dark experimental design

Light and dark responses are fundamental to the biology of plants and produce dramatic differences in gene expression [28]. To verify that the NSF45K array can be used to obtain biologically meaningful data, we performed an experiment to identify rice genes involved in the response of rice to light and dark treatments. With a number of methods as summarized in **Table 1**, we validated our results and the usefulness of the NSF45K array.

For the light vs. dark experiment, we carried out expression-profiling on RNA from leaves of two-week old plants grown in a natural light-dark cycle (light-grown) in comparison to RNA from leaves of plants grown in a natural light dark cycle for a week and then transferred to constant darkness for the second week (dark-grown). In this validation experiment our aim was to identify genes that are universally important to the light/dark response in rice and thus to de-emphasize genotypic differences in the response [31]. Hence, as our biological replicates we employed four different rice varieties with representatives of the two subspecies of rice: *japonicas*, Kitaake, Nipponbare, and Taipei309; and *indica*, IR24. For statistical purposes, we conducted an additional set of hybridizations with the dyes used to label each sample swapped for each genotype (i.e., technical replicates). Details of the experimental design are summarized in **Table S1**.

### Light *vs.* dark treatment was the primary source of variation

To examine sources of variation in our microarray data, we used a diagnostic analysis of variation (ANOVA) method developed by Rocke [32] and demonstrated in Lu *et al.* [33]. We considered the following four sources of variation: the dye incorporated into the hybridization probes (Cy5 or Cy3), sample identity (1–4), treatment (light or dark), and error due to undefined variation. We obtained relative mean square values for the four factors for each spot. Relative mean square was calculated as the mean square of each factor normalized by the sum of mean squares for each spot [33]. The relative mean square value of each factor corresponds to the significance of the factor, with a larger relative mean square value indicating that the factor is more significant.

**Figure 1:**
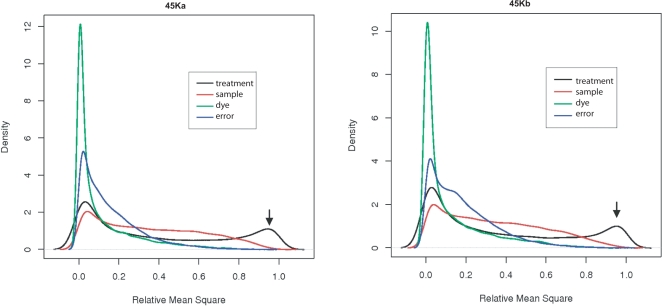
Results of ANOVA analysis to identify the primary sources of variation in the NSF45K light *vs.* dark microarray dataset. Light treatment (treatment, black line), results per each individual slide (sample, red line), whether Cy5 or Cy3 dye was utilized (dye, green line), and inexplicable variation (error, blue line) were the four different factors considered for this ANOVA analysis. The significance of each factor across the NSF45K array was evaluated as a frequency distribution of relative mean square values. The frequency is marked as density on the y-axis. This analysis was carried out separately for the two slides of the array, NSF45Ka and NSF45Kb, as the results could in principle differ significantly. Larger relative mean square values indicate higher significance.

**Figure 1** shows the significance of each factor across the NSF45K array as a frequency distribution (density) of relative mean square values. For both slides, NSF45Ka and NSF45Kb, the treatment (black line) has the largest effect among the factors examined. The next most significant factor is the sample, consistent with the use of different genetic backgrounds [31]; whereas, the dye and random error effects are smaller yet. This analysis indicates that the observed significant changes in gene expression are largely due to the treatment and not to variability in the other parameters examined. This analysis effectively checks the quality of the array data prior to generating a list of genes with significant expression changes.

### Identification of differentially expressed transcripts and independent experimental validation of the microarray results

We used the LMGene Package developed by Rocke [32] to analyze the data and reveal differentially expressed genes between light and dark treatments. This software package has the advantage of being able to identify differences in expression levels even when the expression levels (signal) are low [32]. LMGene uses an empirical Bayseian methodology that combines spot-specific and global error analysis to estimate a false discovery rate (FDR) for each spot. A detailed description of the data generated in this experiment, including average normalized spot intensity, and fold changes in light vs. dark treatments, plant gene ontology (GO) slim terms, and digital northern data based on the number of ESTs in 19 tissue samples (http://www.ricearray.org/rice_digital_northern_search.shtml) can be found in **Table S2**.

As is typical for microarray data, increased confidence is associated with higher fold-change values and greater average signal intensity between the two channels. The distribution of the NSF45K array data, including the relationships between signal intensity, fold change (log_2_ [light/dark]), and FDR are shown in **Figure S3**, **Table S3** and **Table S4**. The signal-to-noise of many oligos with a high level of normalized spot intensity is less; therefore, highly and differentially expressed RNAs are generally associated with lower FDR-values (**Figure S2**). Greater log_2_ (light/dark) values are also associated with lower FDR-values (**Figure S3**). For example, oligos with an FDR of ≤10^−6^ all show nearly 2-fold changes in gene expression or greater (−0.89≤log_2_ [light/dark]≥0.93; **Table S3** and **Figure S2**). Significantly, these trends mean that genes expressed at low levels, such as those that encode many transcription factors, receptor-like kinases, and other regulatory proteins, may undergo biologically significant changes that are not highly statistically significant. We suggest that for genes with lower levels of expression a researcher may need to reduce the fold change-threshold and increase the FDR-threshold when identifying a significant gene list.

Of relevance to further discussion of the data in this manuscript, the list of significantly changing transcripts derived using an FDR of ≤10^−4^ contains 4,962 oligos (**Table S2**). Eighty three percent of oligos on the NSF 45K array correspond to a single transcript (gene model), and the remaining oligos represent genes associated with multiple transcripts [23]. Of the 4962 transcripts, 70% show at least 2-fold induction, or 2,073 and 1,430 transcripts in the light and dark, respectively. Most of these transcripts with a 2-fold change in expression are associated with the even more stringent FDR of ≤10^−6^ (1,695 and 1,098 induced in the light and in dark, respectively). Among transcripts with an expression change of at least 2-fold and a lower confidence (FDR>10^−4^), only 267 and 361 were induced in the light and dark, respectively (**Table S2**). Though most discussion here will focus on transcripts associated with a higher confidence level, many oligos associated with lower confidence changes may still be biologically relevant.

To experimentally verify a subset of the microarray data, we examined the expression patterns of 30 highly light-induced genes by reverse transcription (RT)-PCR (**Figure S1**). FDR-values for these genes were ≤10^−4^, with the exception of one, *Os08g33820.1*. **Figure S1** shows that in all four genetic backgrounds the assayed genes are clearly more highly expressed in the light. Our RT-PCR results provide evidence that the NSF45K array can be used to accurately identify many differentially expressed genes.

### Overview of Gene Ontology analysis

Gene Ontologies (GO) provide controlled vocabulary to describe the biological process, molecular function, and component of the cell to which a gene product putatively contributes [34]–[39]. GO are useful for identifying biological patterns in a list of genes in a genome, microarray data set, or cDNA collection. The terms in GO are linked by a simple, class (parent)–subclass (child) relationship, in which a more specialized, child term can have many less specialized, parent terms. A simplified plant classification system, Plant GOSlim, has been applied to rice gene products (http://rice.plantbiology.msu.edu/GO.retrieval.shtml) based largely on homology with *Arabidopsis* proteins, which have been subject to relatively high quality annotation [16], [22], [34]. The “slimmed” GO provides a broad overview of the ontology content without the detail of many specific, fine-grained terms. Of the oligos on the NSF45K array, approximately 41% have a Plant GOSlim assignment; 28% of assignments are to a biological process, 37% to a molecular function, and 18% to a cellular component (**Table S5**). Many genes have GOSlim terms in more than one category.

To further validate our data and identify processes and cellular compartments most relevant to light and dark responses, we examined the distribution of Plant GOSlim terms within our data. Often, researchers enumerate GO classifications of a microarray-derived gene list selected using a particular p-value or fold-change threshold [40]–[42]. We have used two approaches to improve on this basic analysis. First, we evaluated enrichment or depletion of a GO term in a gene list relative to the frequency of that term in the whole genome with corresponding hypergeometric p-values, using a procedure similar to published methods [43]. To our knowledge, this is the first application in rice of this method of GO analysis. Second, since selection of a particular p-value or fold change is arbitrary and may conceal trends in the data, we evaluated GO term enrichment in data sets delimited by a number of FDR-value thresholds. Johns and Mao [44] used a similar approach to describe the degree of polymorphism among genes in the *japonica* and *indica* genomes by conducting FDR tests with different thresholds.

**Figure 2:**
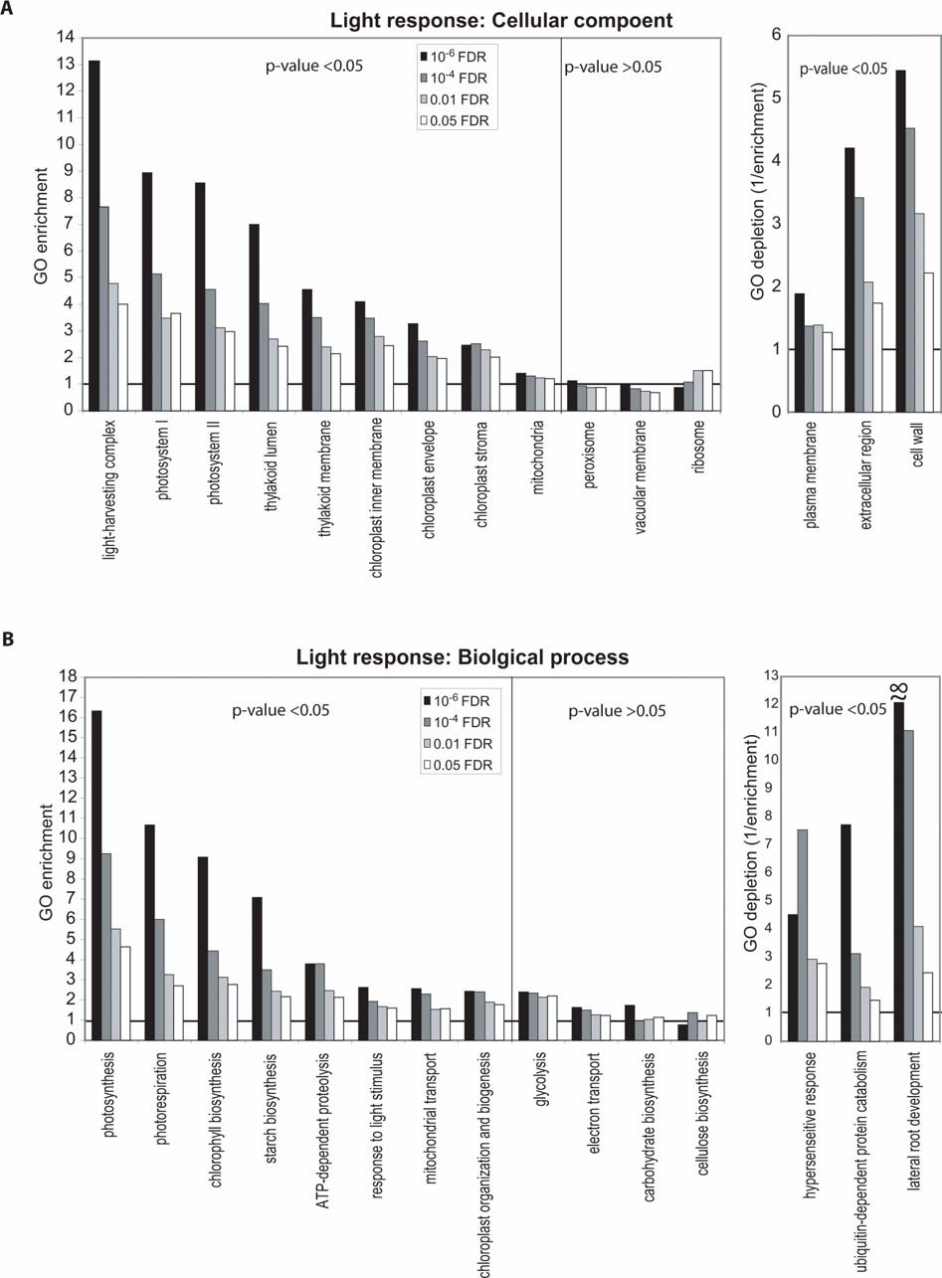
Plant GOSlim enrichment analysis of light-induced transcripts. (A) Enrichment or depletion among light-induced transcripts of selected cellular component GOSlim terms. (B) Enrichment or depletion among light-induced transcripts of selected biological process GOSlim terms. Enrichment and depletion values were generated for transcript lists determine with four FDR cutoff-values, ≤10^−6^ (black bars), ≤10^−4^ (dark grey bars), ≤0.01 (light grey bars), and ≤0.05 (open bars). Shown are selected enriched GOSlim terms with a hypergeometric p-value<0.05 at FDR≤10^−6^ and most other FDRs examined (left panel), less consistently enriched GOSlim terms with a hypergeometric p-value>0.05 at FDR≤10^−6^ (middle panel), and depleted GOSlim terms with a hypergeometric p-value<0.05 at FDR≤10^−6^ and most other FDRs examined (right panel). The y-axis indicates the GOSlim fold- enrichment or depletion. GO enrichment values are calculated as the observed number of transcripts for a particular term divided by the expected number of transcripts and GO depletion values are the inverse of GO enrichment values. A GO enrichment value >1 means that the analyzed term occurs more frequently than expected in a gene list at a selected FDR than it would in a random list with the same number of genes. GO enrichment data for all other GOSlim terms are available in Table S6. The symbol (∞) indicates that the denominator for generating the GO depletion values was zero.

**Figure 3:**
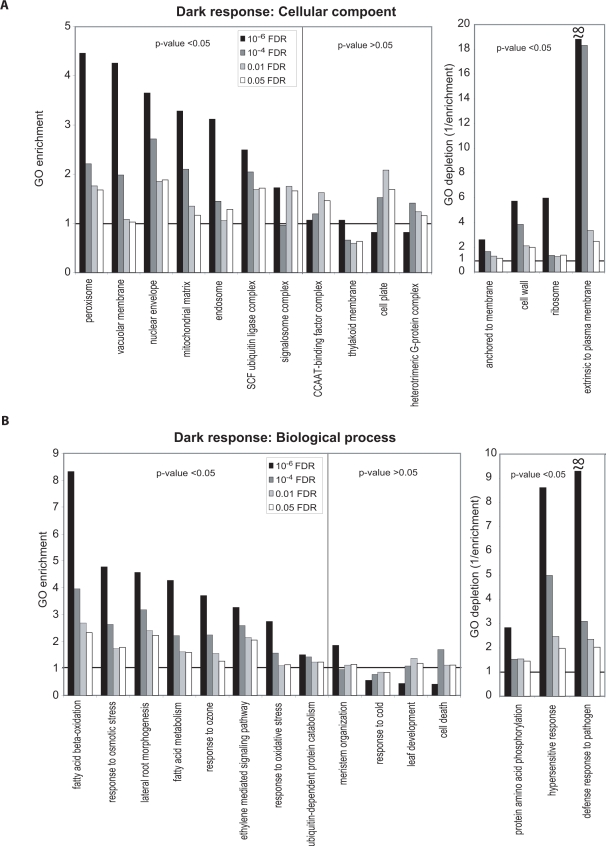
Plant GOSlim enrichment analysis of dark-induced transcripts. (A) Enrichment or depletion among dark-induced transcripts of selected cellular component GOSlim terms. (B) Enrichment or depletion among dark-induced transcripts of selected biological process GOSlim terms. See the Figure 2 legend for a description.

**Figures 2** and **3** show the results of our analysis of enrichment or depletion of fifteen selected biological process and cellular component GOSlim terms among dark and light accumulating transcripts at four FDR thresholds. Other data related to the GO enrichment analysis are available in **Table S6** and **Figure S4**. GO fold-enrichment values of >1 indicate that a GO term occurs more frequently in a gene list compared with the frequency of the term in the genome and suggests that the GO term is representative of a coordinated change in transcript accumulation relevant to the process examined. Conversely, GO enrichment of <1, which can also be expressed as fold-depletion (1/fold-enrichment), indicates that the GO term occurs less frequently in the list than a random list and may indicate general lack of or reduced participation of certain processes or compartments. Based on the hypergeometric distribution, we calculated the probability (p-value) of randomly observing each GO term enrichment or depletion value [43], [45]. This method is one of several that facilitate distinguishing statistically significant patterns in data [46]. **Figure 2**, **Figure 3** and **Figure S4** contain a subset of significantly enriched or depleted GOSlim terms based on their biological meaning, consistency of enrichment or depletion in gene lists determined with different FDR-values, and hypergeometric p-value.

### GO enrichment in the light

Much of the GO enrichment data for light responsive genes is in good agreement with our expectations and provides strong support that the NSF45K array data are valid (**Figure 2** and **Figure S4A**). In the cellular component category, transcripts for the “light-harvesting complex” have the highest level of enrichment among light-induced transcripts (**Figure 2A**). As strongly expected, this and several other plant GOSlim terms associated with chloroplasts show significant enrichment that is maintained in gene lists determined at every FDR-threshold examined (**Figure 2A**). Notably, the GOSlim term “mitochondrion” provides an example of a term that shows only a small numerical fold-enrichment value (1.3-fold at FDR≤10^−6^; **Table S6**). However, because this term is associated with a large numbers of transcripts (1187) the enrichment is highly significant (p = 0.0016 at FDR≤10^−6^), consistent with the role of this metabolic compartment in light-grown plants. On the other hand, the terms “extracellular region”, “plasma membrane”, and “cell wall” are all depleted in the light, with this pattern maintained in gene lists determined with all FDRs (**Figure 2A**; **Table S6**).

For light-induced transcripts, analysis of the biological process principle GO category revealed significant enrichment of GOSlim terms related to known light responses, including “photosynthesis”, “photorespiration”, and mitochondrial and chloroplast anabolism terms. These terms were all associated with hypergeometric p-values of <0.05 and are representative of previously described light-responsive processes [47]–[51]. Conversely, GOSlim terms such as “hypersensitive response” and “lateral root development” are significantly depleted from the light-induced lists, as expected for healthy, above-ground tissues (**Figure 2B**).

### GO enrichment in the dark

Responses of plants to darkness are both less well-studied and less conserved compared with light responses [28]. Figure 3 shows selected GOSlim terms in the principle categories of cellular component and biological process that are enriched and depleted among transcripts induced in the dark relative to the light. Many of the cellular component and biological process GOSlim terms that are enriched in dark-responsive transcript lists are indicative of stressed plants that are in the process of recycling their constituents for survival. For example, “fatty acid beta-oxidation”, which is among the dark-enriched biological process terms, functions to mobilize carbon reserves for use by seedlings when photosynthesis is unavailable [52]. Plant fatty acid beta-oxidation is exclusively peroxisomal [52], [53], which at least partially explains the significant enrichment of this cellular component term in the dark-enriched gene lists. The GOSlim terms “ubiquitin-dependent protein catabolism” (biological process) and “SCF ubiquitin ligase complex” (cellular component) are also enriched among dark-induced transcripts (**Figure 3B**). The Arabidopsis ubiquitin-dependent protease, AtCOP1 degrades a number of transcription factors that function in light responses [54]. Enrichment of this GOSlim term in the dark is consistent with functional conservation of the rice COP1-related gene products and other related proteins.

Other biological process terms related to stress, including “response to oxidative stress” and “response to osmotic stress” were also enriched among dark-induced transcripts. Similarly, four Arabidopsis oxidative stress-related genes are induced during prolonged dark treatment [55]. Consistent with enrichment of mitochondrial and peroxisomal terms in dark-grown rice, peroxisomes, mitochondria, and to a limited extent, chloroplasts, contain multiple enzymes or enzyme systems for removing reactive oxygen species [53]. Furthermore, osmotic and cold stresses have overlapping responses in some cases [56]. However, while “response to osmotic stress” shows significant enrichment in the dark, “response to cold” is only mildly enriched, with the number of terms represented similar to that expected from in random gene lists (**Figure 3B**). We conclude that dark-induced stress overlaps more significantly with specific categories of abiotic stresses, and not abiotic stress in general.

### GO-enrichment analysis at multiple FDRs

**Figures 2**, **3** and **S4** display GO term distributions in transcript lists determined with multiple FDR-value thresholds. To demonstrate the utility of the method, these figures also include a few GOSlim terms that do not show consistent levels of enrichment or depletion in lists determined with various FDRs (middle panels). All terms in these middle panels are associated with high hypergeometric p-values (≥0.05). For example among light-induced transcripts, the term ribosome shows significant enrichment in light-induced lists delimited with FDRs of ≤0.01 and ≤0.05, but as the gene list shrinks with increased confidence, ribosome-associated genes are no longer observed more often than randomly expected (**Figure 2A**). This indicates that ribosome-associated transcripts may only mildly accumulate in the light compared with the dark. Among dark-induced genes, the term “thylakoid membrane” is slightly enriched in the ≤10^−6^ FDR-delimited list but significantly depleted in lists with other FDRs (**Figure 3A**). Among the three highly expressed thylakoid genes that lead to enrichment at ≤10^−6^ FDR is the *copper-transporting ATPase 3* (*APP2*, *Os02g10290*). In Arabidopsis, this protein transports copper ions across the thylakoid membrane and is involved in activation of chloroplastic copper/zinc superoxide dismutase [57], consistent with active oxidative stress protection functioning in dark-stressed rice plants. This example illustrates how GO enrichment analysis for terms with relatively small numbers of assigned genes can be subject to noise. Thus, though the thylakoid membrane is generally involved in light-related, but not dark-related processes, a small, specific subset of thylakoid membrane-localized gene products are dark-related. These examples demonstrate how interpretation of GO enrichment data can depend on the FDR criteria chosen, and thus supports analysis with a range (or a continuum) of criteria to distinguish trends from noise [46].

### Light-responsive genes are abundantly represented among leaf ESTs

To explore and further validate our NSF45K microarray data we compared the results with publicly available rice gene expression data. We developed new tools to facilitate such comparisons in rice, which are available through the rice genome annotation website (http://rice.plantbiology.msu.edu/) for public use. The first of these tools is the Oligo and EST Anatomy Viewer, http://www.ricearray.org/rice_digital_northern_search.shtml (**Table S7** and **S8**), which supports semi-quantitative analysis of EST-based expression profiling data in 19 rice organs and tissues for all loci represented on the NSF45K array (i.e., digital or electronic northern). Digital northern data based on ESTs have corroborated microarray data in other recent studies [29]. Estimated expression patterns from digital northern data have been found to be well matched with RT-PCR data [58].

**Figure 4:**
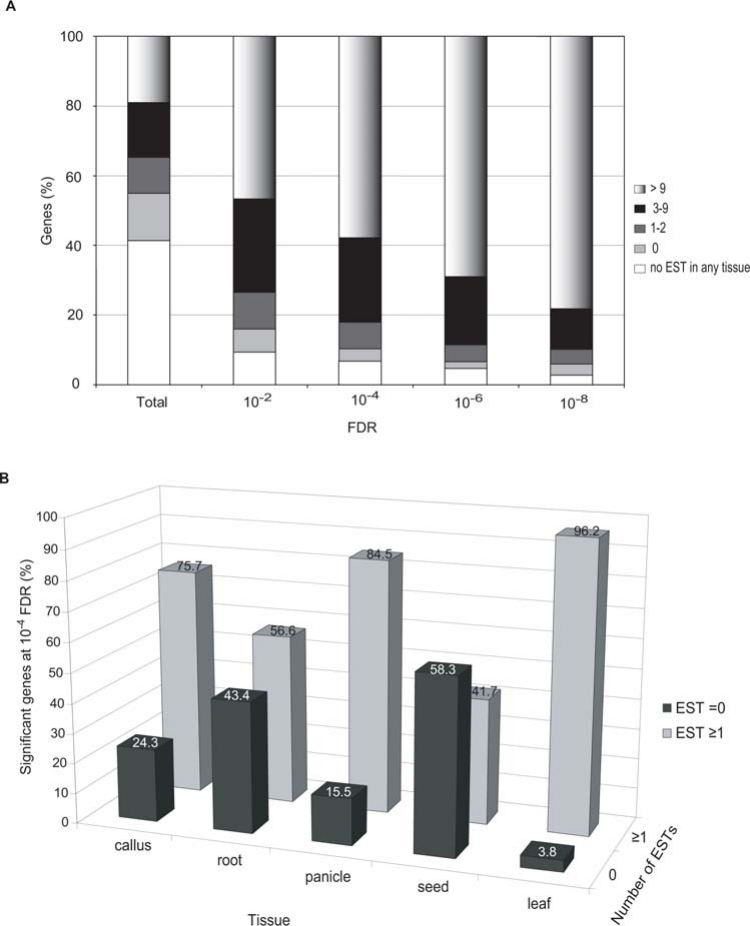
Comparison of microarray data and digital northern data based on ESTs. (A) Percentage of oligos on the NSF45K array with representation in EST leaf libraries or among all EST libraries (total) and among light-responsive transcripts determined at FDRs of ≤0.01, ≤10^−4^, ≤10^−6^, and ≤10^−8^. Bars depict representation by >9 (grey gradient), 3–9 (black), 1–2 (dark grey), or 0 (light grey) ESTs in leaf tissues or no ESTs in all analyzed tissues (white). (B) Percentage of light-responsive transcripts with an FDR of ≤10^−4^ with EST expression levels in leaf, root, panicle, callus, and seed. For this analysis, we included only the oligos on the NSF45K array that correspond to one or more EST in any tissue, i.e., ∼90% of genes with an FDR≤10^−4^. The x-axis indicates the tissues analyzed; y-axis indicates relative percentage of genes having significantly altered expression in light versus dark conditions at a FDR of ≤10^−4^, and z-axis indicates the number of ESTs identified in each tissue, 0 (black) or >1 (grey).

In validation of the NSF45K array data collected on young above-ground tissues, we found that differentially accumulating transcripts associated with greater confidence (i.e., lower FDR) were more likely to appear multiple times in leaf-derived EST collections (**Figure 4A**; **Table S7**). This analysis included both light-induced and dark-induced genes from the NSF45K array. Similarly on the array, differentially expressed transcripts associated with greater confidence were usually more highly expressed (**Figure S2** and **S3**). However, this analysis strongly suggests that most, but not all, transcripts involved in light responses have been captured in EST libraries. Even with an FDR≤10^−4^, about 6.8% (338/4962) of differentially accumulating transcripts lack EST support.

To further examine whether light-responsive transcripts are likely to be associated with gene expression specifically in leaves, we examined the representation of light- and dark-induced transcripts with an FDR≤10^−4^ among ESTs from the following diverse rice organs: leaf, seed, root, panicle, and callus. As shown in Figure 4B, a higher percentage of transcripts that are differentially regulated on the array have EST-support in leaves compared to other organs. For leaves, 96% of differentially expressed oligos have one or more ESTs; whereas, only 4% have one or more EST from another tissue, but not from leaves. Among ESTs from seeds and roots, only 42% and 56%, respectively, of differentially regulated gene products are represented, consistent with these organs not normally being exposed to light. We found that these qualitative patterns of organ specificity also emerged at other FDR-values (data not shown). These results support the hypothesis that light-responsive genes are more likely to be specific to leaves, rather than commonly expressed among leaves and other tissues, such as roots.

### The Rice Multi-platform Microarray Search tool

A major goal of microarray analyses is to identify genes with necessary functions in a particular process that are good candidates for further functional studies. Here, as in many studies, the list of candidate genes with highly significant changes in expression of relatively large magnitude is too extensive for rapid analysis. We took advantage of the >800 rice hybridizations housed at the National Center for Biotechnology Information (NCBI) Gene Expression Omnibus (GEO, http://www.ncbi.nlm.nih.gov/geo/) to further validate our results with the NSF45K array and refine the list of genes likely to be required for rice light responses. Recently, the MicroArray Quality Control (MAQC) project determined that there was a high level of consistency in data across different array platforms [59]. In view of this and the dramatic nature of the response of plants to light, we reasoned that it should be possible to identify genes that show similar expression patterns from public rice array data sets regardless of variations among experimental design, such as cultivar, sample preparation and array platform. With the caveat that gene products with roles in the light responses of specific tissues or growth stages might be missed, such variation might assist identification of consistently responsive genes. However, due to varying platform designs and annotation methods, comparing data from different array platforms has been cumbersome. To solve this, we mapped the oligos from the Affymetrix, Agilent 22K, and YALE/BGI, and NSF45K platforms to the Osa1 Version 5 rice genome annotation gene models and developed the Rice Multi-platform Microarray Search tool (http://www.ricearray.org/matrix.search.shtml). This tool enables users to search across these four different rice oligo microarray platforms to determine which probes from each platform map to a common Osa1 Version 5 rice genome annotation locus.

### Candidate gene list refinement with publicly available array data

To refine the NSF45K light-induced gene list, we selected relevant whole genome microarray datasets (**Table S9**). Most comparable to this current study, Jiao and associates [28] reported on rice gene expression under various light wavelengths using the YALE/BGI array. No light /dark treatment data were available for the Affymetrix array. However, since EST analysis (**Figure 4**) showed that significantly light-induced transcripts are likely to be leaf-expressed, we reasoned that transcripts preferentially expressed in rice seedling tissue compared, for example, to root tissues would be more likely to be involved in light responses. Thus, we judged that the data of Jain and colleagues [29] collected with the Affymetrix array for various stages of development and organs, including young seedling tissue, would also be relevant to this study, which was conducted with 2-week-old seedlings. We also used a number of BGI/Yale data sets gathered for different developmental stages and organs [24], [25] and Affymetrix data sets available for leaves of plants treated with various abiotic stress treatments. Altogether, we identified 39 publicly available array data sets, 19 from the YALE/BGI platform and 20 from the Affymetrix platform for comparison with our data. To compare the two-channel microarray data with single channel Affymetrix array data, we divided each hybridization by a relevant comparison hybridization (**Table S9**). For example for developmentally derived Affymetrix data, we divided the data for young seeding tissue by data for other tissues (e.g. log_2_ [young seedling/root]). We then normalized the resulting ratios to give a similar level of differential expression in comparison with the two channel array data.

**Figure 5:**
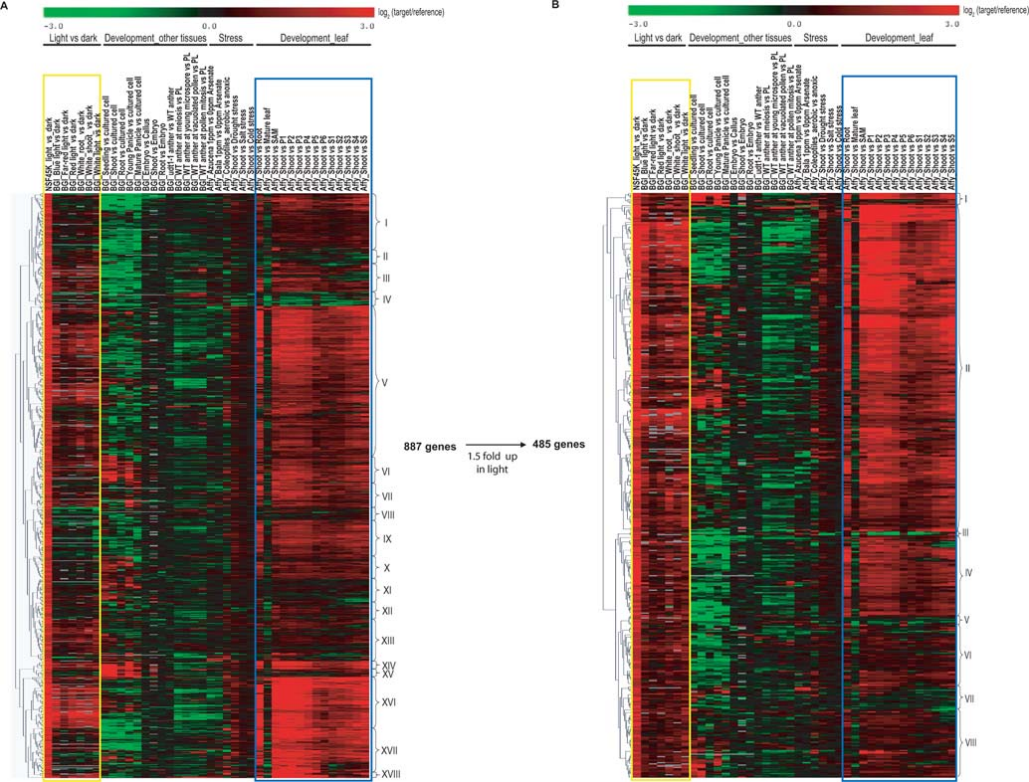
Hierarchical clustering analysis of light-inducible transcripts in 40 rice array data sets gathered on multiple array platforms. (A) Hierarchical clustering analysis of 887 light-inducible transcripts. Included are transcripts from the NSF45K light vs. dark microarray data with >3.1-fold induction in light and FDR≤10^−4^ and which are reported on by all three whole genome rice microarrays. Roman numerals delineate eighteen transcript clusters with similar expression patterns. For hierarchical clustering analysis, we used average log_2_ ratios. In the case of the Affymetrix array data, we downloaded raw data from NCBI GEO and normalized the intensities of all array data (See Materials and Methods). The resulting values were transformed to ratios by comparing with a reference data set followed by log_2_ transformation of the resulting ratios. For BGI/Yale array data, we downloaded from NCBI GEO data in the form of log_2_ ratios of target samples relative to reference samples. We used the average log_2_ ratio for multiple replicates for each condition. (B) Hierarchical clustering analysis of a refined list of 485 consistently light-inducible transcripts. Roman numerals delineate eight clusters with similar expression patterns. Yellow boxed regions indicate the data from light *vs.* dark experiments, and blue boxed regions indicate Affymetrix data for expression patterns relative to young seedling tissue.

Having standardized the data across the three microarray platforms, we selected 887 transcripts from our data that showed ≥3.1-fold induction in the light with an FDR≤10^−4^ and that were present on the all three (near) whole genome microarray platforms. We used the TIGR Multiexperiment Viewer (MeV, http://www.tm4.org/mev.html) to perform an unsupervised, hierarchical clustering analysis of the expression of the 887 transcripts across the 40 microarray data sets [60]. This analysis resulted in 18 transcript clusters with similar differential expression patterns (**Figure 5A**). We characterized these clusters for patterns with respect to consistent light induction (red in datasets delimited with yellow box) and/or consistent induction in young seedlings relative to other tissues (red in datasets delimited with blue box). Several transcript clusters were highly induced in both the light and seedling leaves, including clusters III, V, VI, VII, XIII, XVI, and XVIII, with cluster XVI containing the most highly induced transcripts. Transcripts in clusters X, XI, and XIII showed more moderate differential expression in either the light or in leaves compared to the previously mentioned clusters. The remaining clusters were not consistently induced in either light, young seedlings or both. To identify consistently light-induced transcripts, we applied the criteria of >1.5-fold induction in at least three of the seven light *vs.* dark data sets generated using the NSF45K and YALE/BGI array platforms [28] and narrowed the list of transcripts approximately by half to 485 (**Table S10**).

### Evaluation of the refined light-induced transcript list

Hierarchical clustering of the refined set of 485 transcripts results in eight major clusters (**Figure 5B**). Most of this refined set of consistently light-induced transcripts, except a small number of transcripts in cluster III, V, and VII, also exhibit preferential expression in young seedlings compared with other developmental stages (**Figure 5B**). This finding is consistent with the result that leaf-expressed genes are more likely to be differentially regulated in response to light (**Figure 4**). We also compared the representation of “chloroplast” and “mitochondrion” cellular component GOSlim terms between refined and unrefined lists. In the refined transcript list, there are 204 gene products with a GO term in the cellular component category. Approximately 80% (164/204) have GO terms related to “chloroplast” and “mitochondrion”. While in the unrefined transcript list, about 65% (217/341) of gene products with a cellular component assignment are related to “chloroplast” and “mitochondrion” (data not shown). Enrichment of these terms in the refined list demonstrates the effect of refinement toward focusing the list to transcripts that are consistently involved in light responses.

Changes in the distribution of the light-related GO biological process terms, “photosynthesis” and “photorespiration,” compared with terms not directly related to light shows a significant enrichment of light-related terms relative to unrelated terms after refinement. From a total of 32 “photosynthesis” GOSlim assignments in the rice genome, 17 and 15 gene products are found in the original and refined lists, respectively. Thus, while refinement reduces the list size by approximately half, most of the photosynthesis-related genes are retained. A similar pattern occurs with the GOSlim term, “photorespiration”. Out of 17 gene products with this assignment in rice, seven and six transcripts are found in the original and refined lists, respectively. In contrast, representation of transcripts with the GOSlim terms not directly involved with light responses, such as, “response to wounding” and “response to heat”, is proportional to the size of the lists. Of the 226 “response to wounding” GOSlim terms in the genome, seven are present in the unrefined list; whereas, three are in the refined list. Similarly, of the 212 “response to heat” GOSlim terms, eight and four are in the unrefined and refined lists, respectively. This analysis demonstrates that genes that are consistently light-inducible are more likely to be meaningful and emphasizes the power of integrating multiple microarray data sets for functional genomics analysis, whenever possible.

In summary, we developed the Rice Multi-platform Microarray Search Tool to integrate data from other rice microarray platforms with those from the NSF45K array. Further, we narrowed the candidate transcript list by considering consistency of expression patterns of the most relevant publicly available data sets. In the next section, we will utilize this analysis of consistent induction in multiple data sets generated from different array platforms to facilitate analysis of putatively redundant gene products in a biochemical pathway.

### Biochemical pathway analysis based on gene expression

As a primary functional validation of the biological relevance of the data generated with the NSF45K array, we conducted a finer analysis of a light-regulated biochemical pathway, namely photorespiration. Integration of gene expression data with pathway information has revealed that often gene products that function in the same pathway have similar gene expression patterns. For example, genes encoding the Arabidopsis tetrapyrrole biosynthesis pathway were intensively analyzed using a mini-microarray consisting of 35 genes [61]. Most of steps of this pathway were found to be induced by light. Other rice microarray studies have also revealed involvement of biochemical pathways in various processes. Jiao and associates found that light induces numerous central anabolic pathways, including purine nucleotide biogenesis, gluconeogenesis, and leucine and valine biosynthesis [28]. In another example, salt treatment induced anthocyanin synthesis in a salt sensitive cultivar relative to a salt tolerant cultivar [26], [28]. Those studies highlight that data can be applied to carry out functional study of the participation of particular pathways in various biological processes. Here, we analyze expression of transcripts for a single biochemical pathway toward assigning roles for putatively functionally redundant genes for that pathway.

### Refinement of the candidate gene list for photorespiration

**Figure 6:**
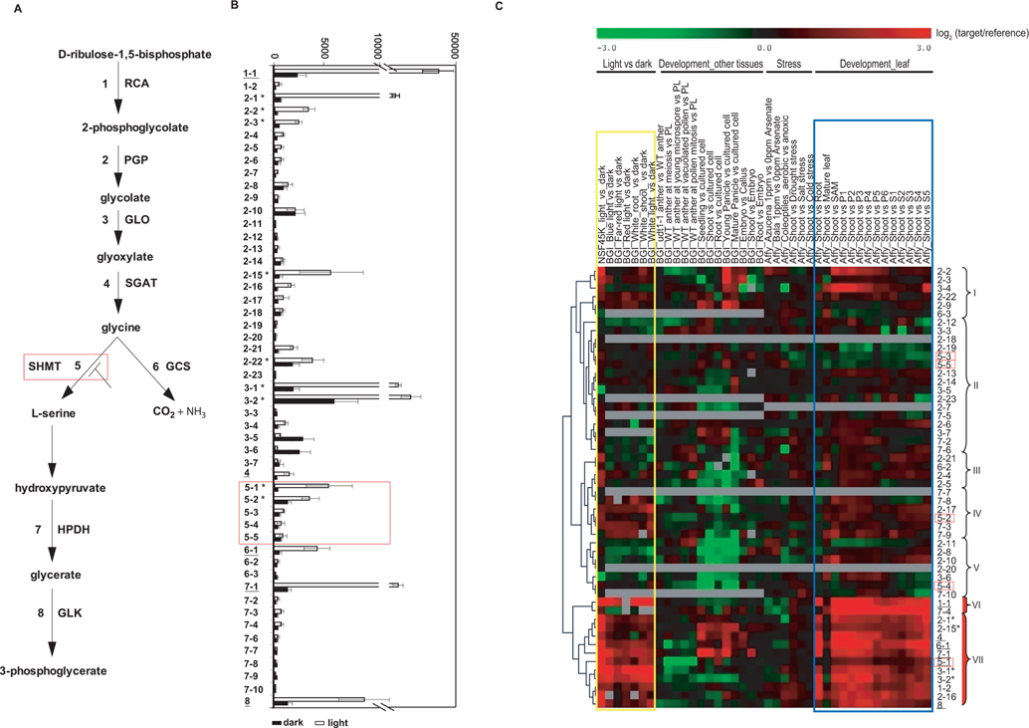
Analysis of the 52 genes predicted to encode enzymes involved in eight steps of the photorespiration pathway. (A) Depiction of the rice photorespiration pathway as provided in the RiceCyc pathways in Gramene (http://www.gramene.org/pathway/). Step 1 is catalyzed by ribulose-1,5 bisphosphate carboxylase, which is regulated by rubisco activase (RCA) 1 [63], [70], [85] for which there are two gene family members (1-1 and 1-2). Rubisco oxygenase activity results in the two-carbon molecule, 2-phosphoglycolate. 2-phosphoglycolate is converted to glycine by phosphoglycolate phosphatase (Step 2), glycolate oxidase (Step 3) and glycine aminotransferase (Step 4). There are 23 gene family members for phosphoglycolate phosphatase (PGP; 2-1 to 2-23). Glycolate oxidase (GLO) has seven gene family members (3-1 to 3-7). Serine-glyoxylate aminotransferase (SGAT) is a unique gene (4). The decarboxylation of two glycines by the glycine cleavage system (GCS; Step 6) generates serine, CO_2_ and NH_3_. Serine is further converted to 3-phosphoglycerate by serine hydroxymethyltransferase (SHMT; Step 5), hydroxyacid dehydrogenase (HPDH; Step 7), and glycerate kinase (GLK; Step 8). There are five genes for SHMT (5-1 to 5-5). There are three GCS gene family members (6-1 to 6-3); Ten d-isomer specific 2-HDPHs (7-1 to 7-10). GLK is a unique gene (8). The three-carbon molecule, 3-phosphoglycerate generated from photorespiration re-enters the CO_2_ fixation Calvin cycle [86]. The SHMT (Step 5) gene identified in this study for which a mutant gives a defect is in the red box. (B) Average expression levels of the 52 candidate photorespiration pathway genes in the NSF45K light vs. dark data set. Open bars show the average absolute signal intensity in the light of four replicates; black bars show the average absolute signal intensity in the dark of four replicates. Underlining indicates genes that are unique or predominantly expressed compared to other gene family members. Asterisks indicate genes that have high expression, but with levels that do not clearly predominate over other gene family members. (C) Hierarchical clustering analysis of the differential expression patterns of the 52 candidate photorespiration pathway genes carried out using 40 microarray data sets. The yellow box delimits data from light *vs.* dark experiments; the blue box indicates Affymetrix developmental data compared to young seedling tissue. Clusters VI and VII (red-filled brackets) have more significant gene expression patterns in NSF 45K and BGI/Yale microarray data in response to light. Underlined gene labels indicate unique or predominantly expressed genes in the photorespiration pathway, based on the consistency of light induction and preferential expression patterns in young seedling tissue. Asterisks indicate gene family members that fall within the same cluster and are expected to have functional redundancy. Genes in step 5 are marked with red open boxes. The microarray data corresponding to the putative photorespiration genes are provided in Table S11.

As displayed in Figure 6A, the photorespiration pathway recovers the product of the ribulose bisphosphate oxygenation by ribulose bisphosphate carboxylase/oxygenase (Rubisco). Under conditions of low CO_2_ and high O_2_ concentrations, Rubisco oxidizes ribulose bisphosphate to 2-phosphoglycolate (and 3-phosphoglycerate), leading to loss of a fixed carbon molecule [62], [63]. Global understanding of the photorespiration pathway through functional analyses in rice and other C_3_ crops may provide a way to enhance the efficiency of photosynthesis and improve crop yield [64]. Genes for photorespiration were prominently identified through Plant GOSlim analysis as being highly enriched among gene products that accumulate in the light (**Figure 2B**). Of the eight steps of the photorespiration pathway, genes for six of these steps are among the refined 485 genes found to be consistently light-induced and preferentially leaf-expressed (**Table S10**). Consistent with the prominence of this pathway in the light-induced data, Rubisco activity is low in darkness and increases with light [65].

In rice, 52 genes encode enzymes predicted to catalyze the eight steps in the photorespiration pathway in RiceCyc of Gramene (http://www.gramene.org/pathway/) (**Figure 6A**). Single genes in the rice genome code for the enzymes that catalyze steps 4 (serine-glyoxylate aminotransferase) and 8 (glycerate kinase) (**Figure 6B**). The other six steps are potentially catalyzed by enzymes encoded for by one or more paralogous genes that are closely related to each other by sequence homology, (designated by 2-1, 2-2, and so on). We used rice microarray data to address the following question: do all of these loci contribute equally to catalyzing photorespiration?

NSF45K array expression data show that only one or two genes for each step show higher absolute expression relative to other genes that putatively participate in the same step, i.e., predominate (**Figure 6B**). Following the observation that genes that function in biochemical pathways tend to be coordinately expressed, we hypothesized that the unique and predominantly expressed genes for each step are those that function in photorespiration. Of steps encoded by gene families, there are two genes in step 1, three in step 6 and ten in step 7. Genes 1-1 (*Rca1*), 6-1 (*glycine decarboxylase*) and 7-1 (*D-isomer specific 2-hydroxyacid dehydrogenase*) are the predominantly light-expressed genes in each gene family (**Figure 6B**). On the other hand, steps 2, 3, and 5 exhibit functional redundancy in the light in terms of absolute gene expression patterns. For these steps, two gene family members exhibit relatively high levels of light-induced expression, or co-predominate. For step 2 (phosphoglycolate phosphatase), both 2-1 and 2-15 co-predominate; for step 3 (glycolate oxidase), 3-1 and 3-2 co-predominate; and for step 5 (serine hydroxymethyltransferase), both 5-1 and 5-2 co-predominate according to the NSF45K microarray data (**Figure 6B**).

We have found that consistent gene expression patterns under similar experimental conditions to be a good indicator to refine the candidate gene lists. Specifically, we recently described five genes that were highly induced in the light in the NSF45K array data but not in BGI/Yale light *vs.* dark array datasets. However, functional analyses with homozygous T-DNA insertional lines of these genes has not yet identified defective phenotypes [66]. In view of the apparent benefit of using other microarray data to corroborate and refine gene lists, we also evaluated the differential expression patterns of the 52 putative photorespiration genes across the 40 microarray data sets described above (**Figure 6C**; **Table S11**). Unsupervised clustering of the differential gene expression patterns of the putative photorespiration genes across these data, revealed that genes in clusters VI and VII are mostly highly expressed in young seedlings and are significantly induced in the light (**Figure 6C**). The exception in these clusters is gene 7-4, which displayed inconsistent light inducibility between the two array platforms and low absolute expression according to the NSF45K array; it is excluded from the following analysis. Six genes in the photorespiration pathway, 1-1, 4, 5-1, 6-1, 7-1, and 8, are grouped within clusters VI and VII and apart from other gene family members (**Figure 6C**). In this list, 4 and 8 are unique sequences, while genes 1-1, 6-1 and 7-1 are predominantly expressed gene family members according to the analysis of NSF45K data. This clustering analysis distinguishes 5-1 among the five genes that putatively catalyze step 5; whereas, analysis of the NSF45K array data alone revealed similar absolute expression levels of genes 5-1 and 5-2. When the additional data are considered, 5-1 clearly clusters with the other highly differentially expressed genes in cluster VII, and 5-2 falls into cluster IV with other less differentially expressed genes. Below we describe the functional data that supports the assignment of genes in clusters VI and VII to encode enzymes that function in photorespiration in young seedlings.

Unlike the six steps we predict to be carried out by the products of unique or predominantly expressed genes, neither the NSF45K array data nor clustering analysis with other data sets differentiates between the other co-predominantly expressed loci corresponding to steps 2 and 3 of photorespiration. Genes 2-1 and 2-15 both fall in cluster VII, as do 3-1 and 3-2 (**Figure 6C**) and all of these transcripts show comparatively high absolute levels of expression according to the NSF45K array data (**Figure 6B**). From this result we infer that steps 2 and 3 in this pathway may be catalyzed by the products of two members of the associated gene families. We predict that functional analysis of these genes with single knockout mutants may therefore be impeded due to functional redundancy in which loss of a single gene may be compensated for by expression of the co-predominantly expressed paralog, masking association of either paralog with a phenotype. Further functional studies of these steps may thus require silencing of multiple family members using RNA interference or double knockout mutants. Silencing of multiple genes was recently applied to the rice *OsRac* gene family [67].

### Evaluation of the method used for refinement of the photorespiration gene list

Data regarding rice and Arabidopsis orthologs of predicted rice photorespiration genes support the use of predominant expression and/or highest differential expression for predicting the function of gene products in biochemical pathways. Mutations in the Arabidopsis *RCA1* gene (step 1), *serine-glyoxylate aminotransferase* (*SGAT*, step 4) gene and, *glycerate kinase* gene (step 8) display stunted and chlorotic phenotypes in normal air but the phenotypes are repressed at elevated CO_2_ [62], [68]–[70]. Similarly, a dramatic reduction in growth and bleached leaves was observed in 3-month-old *Rca1* (1-1) under-expressing rice plants [71], [72].

We have recently examined the function of the rice gene serine hydroxymethyltransferase (5-1) and found that it also appears to function in light-dependent processes [66]. This study made use of the currently available indexed knockout mutant collection that now covers ∼50% of rice gene models (http://signal.salk.edu/RiceGE/RiceGE_Data_Source.html) [23]. A T-DNA insertional knockout mutant for gene 5-1 showed a variegated leaf morphology in the early seedling stage [66]. Further, examination of photographs available for a Tos17 insertional mutant collection (NC2658, http://tos.nias.affrc.go.jp) reveals that mutants in this gene show the same phenotype as the rice T-DNA insertional mutant. The phenotype is similar to that of a mutant of the Arabidopsis ortholog of 5-1, SMH1, when it is grown at ambient CO_2_ [66], [73]. Consistent with the Arabidopsis phenotype, *SHM1* displays the highest induction of the five Arabidopsis gene family members in the various light conditions using Genevestigator (https://www.genevestigator.ethz.ch/) (KHJ and PCR unpublished data).

This analysis of the photorespiration pathway highlights the usefulness of incorporating transcriptomics data with pathways to distinguish among multiple gene products putatively involved in metabolic pathways. The absolute expression levels in the NSF45K data alone were able to guide identification of three predominantly expressed genes. Examining differential expression in the NSF45K and other array data pointed toward another candidate gene, which has been supported by mutant analysis of gene 5-1. From the available genetic data, we conclude that unique and predominantly expressed genes from closely related families are excellent candidates for further functional study. Consistent with this, homozygous T-DNA insertion lines in 6 of 11 highly light-inducible unique genes and 4 of 9 predominantly light-inducible gene family members showed defective light-related phenotypes, such as albino, pale green, or growth retardation [66].

We propose that this method can be an effective means to select candidate genes for functional studies with single knockout mutants or for designing effective experimental schemes for functional analyses of candidate redundant genes. However, the general applicability of focusing on predominantly expressed or highly differentially expressed genes involved in diverse processes remains to be determined. As with all studies that use gene expression as an indication of function, this analysis will be unable to identify gene products that are involved in the process under examination but whose transcript levels do not appear to change in general, or at least in the samples examined. Complementary genetic, biochemical or protein-protein interaction studies will be needed to detect such genes/gene products [74], [75].

### Conclusion

Rice is a crop of paramount importance to humanity and has emerged as the primary reference for study of grass genomics. Despite the modern molecular genetic tools available for analysis of the rice genome, the number of fully characterized genes in rice to date lags far behind that of the reference dicot, Arabidopsis. We have developed a publicly available NSF45K oligo array and employed five methods to demonstrate that it can be used to obtain high-quality, biologically relevant data. Advantages of this array include its low cost, availability of detailed information about the oligos spotted onto the platform (http://www.ricearray.org/), and web-based tools for analysis. Application of the methods established in this study for refining lists of candidate genes obtained through transcriptome analyses, including the identification of likely functional paralogs among hypothesized paralogs, will allow for more efficient functional studies of this important plant.

## Materials and Methods

### NSF45K Array and Annotation

The usefulness and accuracy of an oligo microarray platform relies on careful design of the oligos. We used the oligo identification tool, PICKY 2.0, to design the 50- to 70-mer oligos that comprise the NSF45K array [76]. Because species with large genomes tend to contain large numbers of homologous genes, it is not possible to design long oligos capable of differentiating among all genes in these species [30], [76]. It is particularly difficult to differentiate transposable element (TE)-related genes, which amount to 15,424 gene sequences in the rice genome (http://rice.plantbiology.msu.edu/pseudomolecules/info.shtml) [77]. Among the rice TE-related genes, PICKY 1.0 software, for example, was able to identify unique oligos for only a couple of hundred [76]. The improved PICKY 2.0 includes a new feature that groups highly similar genes and designs oligos for the groups, including sets of alternatively spliced isoforms. Using PICKY 2.0, we applied an oligo design stringency requiring less than 17 nucleotides exact match to any non-target and a 10°C minimum separation of hybridization temperature between the highest affinity non-target and the target for all genes (http://www.complex.iastate.edu/download/Picky/Picky2_oligos/RiceOligos.html) [76]. These criteria led to the design of 43,311 oligo probes that target 45,116 gene models out of a total of 61,420 target transcript sequences in the TIGR V3 rice gene set release.

The array is printed on two slides, NSF45Ka and NSF45Kb. NSF45Ka contains 23,040 oligos including 240 oligos complementary to the *hygromycin phosphotransferase* (*hph*) gene (GenBank Accession: AF354045), a selectable marker used in transgenic rice generation. NSF45Kb contains 20,727 oligos including 216 *hph* oligos. The *hph* oligos serve as positive controls for experiments comparing transgenic plants with wild type plants. These show approximately 10-fold induction relative to non-transgenic samples (data not shown). Alternatively, the *hph* oligos serve as negative controls for non-transgenic samples, as in the light vs. dark experiment, in which all of our samples were harvested from non-transgenic plants. Of the 456 *hph* control oligos on the array, there were only three that showed differential expression with ≤0.05 FDR (**Table S12**). This result suggests that the *hph* probes can effectively be used as negative controls. In addition, due to the uniform signal and distinct pattern produced by the *hph* oligos, they help align array matrices with images, avoiding data collection errors due to mis-alignment.

The rice genome annotation project has recently released the Osa1 Version 5 rice genome annotation, and we have mapped the oligos on the four available rice oligo microarray platforms to this latest annotation [23]. The NSF45K array covers 30,797 putative/known genes, 7,182 expressed genes, 1,259 conserved hypothetical genes, and 6,862 hypothetical genes (http://www.ricearray.org/rice_layout.shtml), with 32,975 unique oligos for single gene models (transcripts) and 6,544 shared oligos corresponding to 15,003 gene models [23]. The unique oligos distinguish individual gene models; whereas, the shared oligos are designed to more than one gene model, allowing users to capture data on the corresponding gene families. Importantly, the shared oligos used in combination with the unique oligos can detect alternative splice forms. Of the 43,311 oligo probes on the array, 3,792 did not map to the Osa1 Version 5 rice genome annotation gene models. Of these, 1784 target non-exonic regions of Version 5 gene models due to updated annotation of previous TIGR version 3.0 gene models, 237 map to unanchored models, 300 map to rice genome annotation Transcript Assemblies, 55 map to chloroplast genes, and 44 map to mitochondrial genes.

### Oligo synthesis and array fabrication

Oligos were commercially synthesized by Integrated DNA Technologies (IDT, www.idtdna.com; Coralville, IA). Copies of the *hph* oligo were randomly placed into four wells in each 384-well oligo synthesis plate serving as a quality control for array printing and hybridization.

The contents of the oligo synthesis plates were normalized to equimolar concentrations and lyophilized by IDT. The oligos were then resuspended in sterile water to a concentration of 50 µM in 384-well microtiter plates. The oligos were combined with 1× Pronto!™ Universal Spotting Solution (Corning, Inc., Corning, NY) to a final concentration of 25 µM in 0.5× spotting solution and spotted onto bar-coded Corning UltraGAPS™ γ-amino-propyl-silane-coated microarray slides (Corning, Inc., Corning, NY). Spotting was performed using an Intelligent Automation Systems custom-built arrayer (Brooks Automation, Chelmsford, MA) with a 48-pin print head. Printing was performed under environmentally controlled conditions of 72°C and 45% relative humidity. Oligos were bound to the glass slides by UV-crosslinking at 25 mJ/cm^2^. Completed slides were stored in the dark under argon at room temperature until use. Information about the array platform design has been deposited at the NCBI Gene Expression Omnibus (GEO, http://www.ncbi.nlm.nih.gov/geo/) under the accession numbers GPL4105 and GPL4106.

### Plant growth

Nipponbare, Kitaake, Taipei 309 (TP309), and IR24 rice seeds were germinated by imbibing for three days in the water and subsequently planted in clay soil and maintained in a greenhouse. For light treatments, seedlings remained in the greenhouse for two weeks. For dark treatments, seedlings were moved after 7 days to an incubator (Percival Scientific, Inc., Perry, IA) and maintained in continuous darkness at 28°C for another 7 days. Approximately 50 leaves from 2-week old rice plants were collected for each biological replicate at 2 pm for the light and 11 pm for the dark.

### RNA and Probe preparation

Total RNA was isolated using TRIZOL reagent (Invitrogen, Carlsbad, CA), DNaseI-treated for 15 minutes, purified using RNeasy Midi Kits (Qiagen, Germantown, MD), and enriched for poly-A RNA using the Oligotex mRNA Kit (Qiagen). All steps were performed according to the manufacturer's instructions. The quality of RNA was visualized after electrophoresis through a 1% agarose gel followed by staining with ethidium bromide. The quantity of total RNA and mRNA were determined using a Nanodrop ND-1000 spectrophotometer (Nanodrop, Wilmington, DE). In addition, the level of protein contamination in the RNA was determined based on the A_260_/A_280_ ratio.

We used unamplified mRNA to make probe for microarray experiments, as, when obtainable, unamplified material provides the least bias for transcriptome analysis. Labeled probe for hybridizations with the NSF45K microarray were prepared from 1–2 µg mRNA samples using the SuperScript™ indirect cDNA Labeling System (Invitrogen, Carlsbad, CA). This system utilizes a secondary labeling method and thereby avoids the dye bias commonly associated with direct incorporation of dye-modified nucleotides during the reverse transcription reaction. Amino allyl-dUTP is incorporated during cDNA synthesis followed by coupling of the amino allyl-modified cDNA with a fluorochrome (Cy3 or Cy5). A more detailed procedure follows.

*In vitro* reverse transcription was performed using 1 µg mRNA combined with random 9-mer and oligo(dT) primers with incubation for 3 hours at 46°C in a final volume of 30 µl containing SuperScript™ III Reverse Transcriptase (400 U/µl), 5× reaction buffer, 0.1 M DTT, and a dNTP mixture including amino allyl-modified nucleotide (AA-dUTP). Subsequent to reverse transcription, the RNA template was hydrolyzed using 15 µl of 1 N NaOH (70°C, 15 min) followed by neutralization with 15 µl of 1N HCl. Unincorporated primers and nucleotides were removed using the S.N.A.P.™ Column purification system according to the manufacturer's protocol (Invitrogen) and the purified amino allyl-modified cDNA was re-suspended in 5 µl of the coupling buffer supplied by the manufacturer. The amino allyl-modified cDNA was then mixed with lyophilized Cy3 or Cy5 suspended in 5 µl dimethylsulfoxide (Sigma-Aldrich Corp., St. Louis, MO) and incubated for 1 hr at room temperature in the dark. The reaction was quenched by adding 15 µl of 4 M hydroxylamine (15 min, room temperature in the dark). Dye-coupled cDNA was then purified by using the S.N.A.P.™ Column purification system.

### Microarray hybridizations and scanning

All hybridizations were conducted at the Arraycore Microarray Facility at the University of California, Davis (http://array.ucdavis.edu/home/). Prior to hybridizations, microarrays were treated with a solution containing sodium borohydride to minimize non-specific autofluorescence from the spotted material as described previously [78]. For this, slides were placed into a solution containing 2× saline-sodium citrate (SSC), 0.05% SDS, and 0.25% NaBH_4_ (Sigma, St. Louis, MO) and incubated at 42°C for 20 min. Slides were transferred to 1× SSC for 5 min at room temperature and then sequentially washed with vigorous stirring using fresh 1× SSC (3×5 min, room temperature), 0.2× SSC (4×2 min, room temperature), and Nanopure (Millipore, Milford, MA) water (1×2 min, room temperature). Slides were spin-dried (1000 rpm, 10 min) and stored under argon until use.

Hybridizations were performed in a clean room environment (HEPA- and carbon-filtered) to minimize exposure of microarrays and labeled-targets to dust and ozone [79]. Microarray pre-hybridization, hybridization, and washes were performed using an HS4800 Automated Slide Hybridization Station (Tecan, Switzerland). Corresponding Cy3- or Cy5-labeled cDNA targets were mixed and dried by vacuum centrifugation. Targets were suspended in 100 µl GeneFrames hybridization solution (MWG Biotech, UK), incubated in boiling water for 3 min, centrifuged (14,000×g, 5 min), and kept at room temperature until injection into the hybridization station. Microarray slides were pre-hybridized in the hybridization station for 15 min at 50°C in 5× SSPE, 6M Urea, 0.5% Tween-20, 10× Denhardt's solution (Sigma). Samples were hybridized for 16 hours at 50°C with medium agitation, then sequentially washed in solutions comprising 2× SSC, 0.2% SDS [2×(1 min wash, 1 min soak, 37°C)], 1× SSC [2×(1 min wash, 1 min soak, 37°C)], and 0.5× SSC [2×(1 min wash, 1 min soak, 30°C)], and then dried under N_2_ (5 min, 30°C). Slides were kept under N_2_ until they were scanned.

Hybridized microarray slides were imaged using a GenePix 4000B dual laser microarray scanner (Axon Instruments, now part of Molecular Devices, Sunnyvale, CA) at 5 µm resolution using 100% laser power for both lasers (532 and 635 nm). Slides were scanned twice, once using a high photomultiplier tube (PMT) and again using low PMT settings.

### Microarray data processing and normalization

Spot intensities were quantified using Axon GenePix Pro 4.0 image analysis software. Afterwards, GenePix Pro 4.0 result data files (.gpr files) were generated using high PMT and low PMT settings. For high PMT, we normalized replicated data to minimize the variations caused by experimental procedures using the Lowess normalization method in the LMGene Package in R [32], [80]. We further normalized for signal intensity among different experiments using averages of all the gene signals obtained during individual experiments. In addition, we estimated the background “expression” level based on the signal associated with the *hph* gene. From eight hybridizations of four biological replicates, we detected an average normalized spot intensity for the *hph* oligos of 220±30. We then generated average normalized spot intensities following a common strategy by subtracting average *hph* intensity (220) and adding 2 standard deviations (60) of the average normalized *hph* intensity). To identify differentially expressed genes, we used the method in LMGene, as developed by Rocke (2004). FDRs and fold changes of light over dark were generated and the data within 10^−4^ FDR are represented in **Table S2**. The expression data from these experiments are available through GEO (Accession GSE8261). The Affymetrix raw data was downloaded from NCBI GEO (platform Accession Number is GPL2025). We used MAS 5.0 method provided by R package for Affymerix oligo array to convert probe level data to expression values [81], [82]. The trimmed mean target intensity of each array was arbitrarily set to 500. The data were log_2_ transformed.

### GO term enrichment or depletion

We evaluated enrichment or depletion of GO terms for light induced and dark induced genes in each of the principle GO categories, cellular component, biological process, and molecular function. We calculated fold-enrichment in each principle GO category for each GOSlim term (e.g., photorespiration) for gene lists determined with FDR threshold values of ≤0.05, 0.01, 10^−4^, and 10^−6^. For each term, fold-enrichment is the observed number of genes in the gene list divided by the expected number of genes, given the size of the gene list compared with the whole genome. Fold-depletion of a GOSlim term is calculated as 1/fold-enrichment. **Table S6** contains these calculations for each GOSlim term. We used the hypergeometric distribution to evaluate the probability (p-value) of randomly observing the enrichment or depletion for each GOSlim term [43]. GO terms with a p-value ≤0.05 are marked with asterisks in **Table S6**.

### Digital northern data based on the numbers of ESTs

We used the number of ESTs representing specific transcripts isolated from 19 rice tissue sources (i.e. callus, suspension cells, seedling, leaf, shoot, root, stem, sheath, phloem, panicle, flower, anther, pistil, endosperm, immature seed, mixed tissues, mature seed, whole plant, and unknown samples) to estimate gene expression levels in the different tissues. The EST evidence was analyzed using the Program to Assemble Spliced Alignments (PASA) software, which utilizes a number of alignment programs to maximally align transcripts to the genome as introduced by Haas et al. [83].

### Multiplatform microarray search and data generation

All data were downloaded as a series matrix files from NCBI Gene Expression Omnibus (GEO, http://www.ncbi.nlm.nih.gov/geo/). GEO accession numbers are given in **Table S9**. The series record links together a group of related samples. We used the rice Multi-platform Microarray Search (http://www.ricearray.org/matrix.search.shtml) to determine the oligo identifiers for the light-induced genes from our array data. The matrix file available at the Rice Multi-platform Microarray Search web site (http://www.ricearray.org/matrix.search.shtml) allows easy systematic comparison of data across rice whole genome microarrays. The file contains the oligo identifiers of Affymertrix, YALE/BGI, NSF20K, and Agilent 22K rice arrays. Mapping to the latest annotation revealed that the four publicly available rice oligo array platforms together encompass 80–90% of annotated genes in the rice genome, with substantial overlap among the platforms.

We developed a method for comparing data from the different array platforms. Agilent, YALE/BGI, and the NSF45K use a two-color hybridization method with data presented as log_2_ ratios of the signal from different treatments (e.g. log_2_[dark/light]). Affymetrix chip data, however, are generated using a single color array platform and are presented as absolute expression values. Thus, single channel and two channel array data cannot be directly compared. To compare single channel Affymetrix array data with two-channel data from the other arrays, we divided each data set by a relevant comparison data set (**Table S9**). For microarray data from stress-treated samples, we divided the data in various stress-treatment by the data in untreated reference generated in same experiment. From this, genes preferentially expressed in young seedling tissue or samples treated with various stresses have positive values and are shown in red in **Figures 5** and **6**. As most log_2_ ratios calculated from Affymetrix array data relative to expression in seedling were ∼2-fold greater than those of NSF45K light *vs.* dark array data, we reduced the log_2_ ratios we calculated from the Affymetrix array by 2-fold to make them more comparable to the NSF45K light *vs.* dark array data. In contrast and in justification of this adjustment, YALE/BGI array data was quantitatively similar to the NSF45K dataset without any adjustment.

### Hierarchical clustering of microarray data

We used the TIGR MultiExperiment Viewer (MeV, http://www.tm4.org/mev.html) to carry out clustering analyses of the 40 microarray data sets [60], [84]. We generated tab-delimited files comprising the average log_2_ ratios determined for multiple samples selected from the 40 microarray data sets were and used them as input for these analyses. For unsupervised, hierarchical, clustering, we based the analysis on the Euclidean distance, the difference in log_2_ ratios between two genes, which is the default metric distance used for hierarchical clustering [60]. To further refine the significant gene list, we selected 485 genes showing at least 1.5-fold induction in the light in more than three out of seven light-related microarray data sets (NSF45K light *vs.* dark, YALE/BGI blue light *vs.* dark, YALE/BGI red light *vs.* dark, YALE/BGI far red light *vs.* dark, YALE/BGI white light *vs.* dark, YALE/BGI root white light *vs.* dark, and YALE/BGI shoot white light *vs.* dark) and later referred to 14 Affymetrix microarray data sets comprising developmental-stage type experiments in comparison with young seedling tissue (i.e., young seedling tissue *vs.* root, young seedling tissue *vs.* mature leaf, young seedling tissue vs. shoot apical meristem, young seedling tissue vs. panicle 1 [0–3 cm], panicle 2 [3–5 cm] *vs.* young seedling tissue, panicle 3 [5–10 cm] *vs.* young seedling tissue, panicle 4 [10–15 cm] *vs.* young seedling tissue, panicle 5 [15–22 cm] *vs.* young seedling tissue, panicle 6 [22–30 cm] *vs.* young seedling tissue, young seedling tissue *vs.* developing seed 1, young seedling tissue *vs.* developing seed 2, young seedling tissue *vs.* developing seed 3, young seedling tissue *vs.* developing seed 4, young seedling tissue *vs.* developing seed 5, **Table S9**). BGI/Yale array data showing relative expression levels to suspension cultured cells, sequential expression patterns of wild type anthers relative to palea/lemma, and Affymetrix data comparing stress-treated targets to untreated reference are also used to check gene expression patterns in other conditions (**Table S9**).

### Pathway analyses incorporating gene expression profiling data

The pathways used in this study were developed using Gramene RiceCyc (http://pathway.gramene.org/RICE/class-instancesobjectPathways). Candidate genes in the pathways having evidence of expression, such as expressed sequence tags (ESTs) or full length cDNAs, were available at the above website. Additional candidate genes for which no evidence of gene expression was available were selected based on Arabidopsis best-hit genes homologous to the Osa1 Version 5 rice genome annotation gene models. Probable paralogous gene family members of all candidate genes were checked using the information of all rice paralogous gene family members [66].

## Supporting Information

Table S1 Summary of NSF45K light vs. dark array experimental design.
(0.03 MB DOC)

Table S2 Summary of the light- and dark-induced transcripts with an FDR less than 10−4 from the NSF45K microarray data. In the worksheet labeled “less than 10−4 FDR”, there are NSF45K light vs. dark microarray data with a more than 2-fold change in expression and a FDR less than 10−4. The sheet “more than 10−4 FDR”, contains data for transcripts that showed more than 2-fold change in expression, but a FDR more than 10−4. 2073 transcripts show 2-fold higher accumulation (log2-value>1) in the light compared to dark and 1430 show 2-fold higher accumulation (log2-value<−1) in the dark compared to light. Oligo_id is the name of NSF 45K oligo; Locus_id is the Osa1 Version 5 rice genome annotation gene model; FDR is the LMGene-generated adjusted p-value; log2 (Light/Dark) is log2(average normalized spot intensity in the light /average normalized spot intensity in the dark); Avg_light intensity is the average normalized spot intensity in the light; Avg_dark intensity is the average normalized spot intensity in the dark; std_light is the standard deviation in the normalized intensity in the light of all replicates and std_dark is the variation in the normalized intensity in the dark of all replicates. For each oligo, corresponding EST counts for 19 tissues are shown and are labeled according to the organ/tissue from which the ESTs were prepared, including the following: callus, root, leaf, seedling, sheath, phloem, shoot, stem, flower, panicle, anthers, pistil, endosperm, immature seed, seed, suspension, mixed, unknown, and whole plant. Information about the EST data for each tissue is in Table S8. Sum of total ESTs is the number of ESTs from all tissues related to the corresponding oligo. Full length cDNA is the cDNA accession number available from the Knowledge-based Oryza Molecular biological Encyclopedia (KOME, http://red.dna.affrc.go.jp/cDNA/). GO_id indicates the GO identifier accessible at AmiGO (http://amigo.geneontology.org/cgi-bin/amigo/go.cgi?session=7122b1203889484).
(2.63 MB XLS)

Table S3 Relationship between FDR-threshold values, normalized spot intensity, and minimum log2 (light/dark)-values of NSF45K light vs. dark microarray data.
(0.04 MB DOC)

Table S4 Relationship between FDR-interval values, normalized spot intensity, and maximum log2 (light/dark)-values of NSF45K light vs. dark microarray data.
(0.04 MB DOC)

Table S5 Summary of the number of plant GOSlim assignments for the genes that correspond to the NSF45K oligo set. There are three gene ontology (GO) principles: cellular component, biological process, and molecular function. Forty one percent of the NSF45K oligo set have at least one GOSlim term.
(0.03 MB DOC)

Table S6 GO fold-enrichment values at five FDRs for the NSF45K light vs dark array data set. This table consists of six worksheets, one for each principle category (cellular component, biological process, and molecular function) in the light and in the dark.
(2.69 MB XLS)

Table S7 Relationship between FDR-threshold used to determine a significant gene list and expression level based on the number of ESTs in leaves of the NSF45K light vs. dark data. The number of ESTs in five selected tissues (callus, root, panicle, seed, and leaf) were compared to the FDR values from NSF45K light vs dark data. The data in this table are displayed in Figure 4.
(0.05 MB DOC)

Table S8 Information on the libraries used in Oligo and EST Anatomy Viewer. The Oligo and EST Anatomy Viewer tool (http://www.ricearray.org/rice_digital_northern_search.shtml) is based on EST frequency in nineteen tissues. The library identifiers (id) used for this tool are summarized in this table.
(0.12 MB XLS)

Table S9 Summary of rice microarray data from NCBI GEO used for this study. We used the Rice Multiplatform Microarray Search Tool (http://www.ricearray.org/matrix.search.shtml) to identify oligo identifiers (ids) for genes from the different array platforms. We generated log2 fold-change data for 887 light inducible genes selected from the NSP45K light vs dark data and compared the expression of these genes with that in 20 rice Affymetrix array data sets and 19 BGI/Yale rice array data sets. More detailed information on these data is accessible at NCBI GEO (http://www.ncbi.nlm.nih.gov/geo/) with the GEO accession numbers in this table.
(0.17 MB DOC)

Table S10 Microarray data for the 485 genes refined in Figure 5B. Microarray data were generated from 40 data sets generated on three microarray platforms (Affymetrix, BGI/Yale, and NSF45K). A description of the column headings in this table is contained in Table S9.
(0.26 MB XLS)

Table S11 Microarray data for 52 genes putatively acting in the photorespiration pathway shown in Figure 6C. The rice photorespiration pathway consists of 8 steps, as predicted by the RiceCyc tool, which is curated by Gramene (http://pathway.gramene.org/RICE/server.html). The eight steps are encoded by 52 genes whose involvement cannot be excluded a priori. To help narrow the number of hypothesized genes for each step, we evaluated expression patterns of the 52 genes across 40 rice microarray data sets. The information on the column heads in the table is in Table S9.
(0.07 MB XLS)

Table S12 Error rate of NSF45K light vs dark microarray data according to analysis of hph oligos. The NSF45K array contains 456 hph oligos randomly spotted throughout the two slides of the array. They are designed to anneal to the hygromycin phosphotransferase gene, a commonly used selectable marker for Agrobacterium-mediated plant transformation. In the case in which RNA from plant tissue that lacks this gene is used for the array experiment, there should be no specific annealing to these oligos and they serve as negative controls. We used their average spot intensity to calculate the background level of hybridization.
(0.03 MB DOC)

Figure S1 Thirty highly accumulating light-inducible genes were randomly selected for confirmation with RT-PCR with the same RNA samples applied to the NSF45K array. All tested genes consistently showed higher accumulation in the light (L) compared with the dark (D) in the 4 rice varieties (N, Nipponbare; K, Kitaake; I, IR24; T, TP309), similar to the microarray data. Rice Actin1 and Ubiquitin1 RNAs were used as internal controls [58].
(0.63 MB TIF)

Figure S2 The percent of oligos represented by >1000 (grey gradient), 500–1000 (black), 280–500 (dark grey), 120–280 (light grey) or <120 normalized spot intensity (white) determined using the entire oligo set of the NSF45K array without considering FDR (total) and with genes selected from the NSF45K light vs. dark microarray data with FDR thresholds of ≤0.01, ≤10−4, ≤10−6, and ≤10−8.
(1.66 MB TIF)

Figure S3 Comparison of log2 (light/dark) and (−log10) FDR-values. Blue-symbols represent data from the NSF45K slide a, and red symbols are data from the NSF45K slide b.
(5.68 MB TIF)

Figure S4 Fold-enrichment analysis of GOSlim terms in response to light or dark in the molecular function GO category. (A) GO enrichment analysis in the light. (B) GO enrichment analysis in the dark. See Figure 2 for a description of the panels.
(2.06 MB TIF)
