# Supplementary material for: Refinement of Light-Responsive Transcript Lists Using Rice Oligonucleotide Arrays: Evaluation of Gene-Redundancy
Source: PLoS One. 2008 Oct 6;3(10):e3337. doi: 10.1371/journal.pone.0003337 (PMC2556097; doi:10.1371/journal.pone.0003337)
Supplement: Table S1 — Summary of NSF45K light vs. dark array experimental design. (0.03 MB DOC) [file pone.0003337.s001.doc]

**Table S1**. **Summary of NSF45K light *vs.* dark array experiment.**

| Subspecies | Japonica | | | | | | Indica | |
| --- | --- | --- | --- | --- | --- | --- | --- | --- |
| Biological replicate ^a^  (Varieties) | Nipponbare | | Kitaake | | TP309 | | IR24 | |
| Technical replicate ^b^ | 1 | 2 ^b^ | 1 | 2 ^b^ | 1 | 2 ^b^ | 1 | 2 ^b^ |
| Cy5 | Light | Dark | Light | Dark | Light | Dark | Light | Dark |
| Cy3 | Dark | Light | Dark | Light | Dark | Light | Dark | Light |

^a^ We pooled ~50 leaves for each biological replicate. Each two-week-old seedling has 2-4 leaves.

^b^ RNA of each light-grown and dark-grown biological sample was divided in half for labeling with each dye and independent hybridization (i.e., technical replicates, 1 and 2).
