# Supplementary material for: Refinement of Light-Responsive Transcript Lists Using Rice Oligonucleotide Arrays: Evaluation of Gene-Redundancy
Source: PLoS One. 2008 Oct 6;3(10):e3337. doi: 10.1371/journal.pone.0003337 (PMC2556097; doi:10.1371/journal.pone.0003337)
Supplement: Table S3 — Relationship between FDR-threshold values, normalized spot intensity, and minimum log2 (light/dark)-values of NSF45K light vs. dark microarray data. (0.04 MB DOC) [file pone.0003337.s003.doc]

**Table S3. Relationship of normalized spot intensities of microarray and FDR thresholds in microarray.**

| Gene List | Number of Oligos at each Spot Intensity | | | | | Sum |
| --- | --- | --- | --- | --- | --- | --- |
|  | ≥1000^a^ | 500 – 1000 | 280 – 500 | 120 – 280 | 0 – 120 |  |
| Whole Array | 4341^b^ | 3572 | 3344 | 5,275 | 26,779 | 43,311 |
| FDR ≤ 0.05  (minimum log_2_) | 3513  (0.20^c^, -0.19^d^) | 2498  (0.18, -0.19) | 2099  (0.19, -0.18) | 3,512  (0.18, -0.17) | 1,391  (0.17, -0.17) | 13,013  (0.17, -0.17) |
| FDR ≤ 0.01  (minimum log_2_) | 3220  (0.24, -0.26) | 2196  (0.24, -0.26) | 1750  (0.26, -0.25) | 2,069  (0.25, -0.25) | 1,126  (0.25, -0.23) | 10,361  (0.24, -0.23 |
| FDR ≤ 10^-4^  (minimum log_2_) | 2309  (0.53, -0.49) | 1206  (0.50, -0.49) | 753  (0.46, -0.50) | 600  (0.49, -0.46) | 94  (0.52, -0.47) | 4962  (0.46, -0.46) |
| FDR ≤ 10^-6^  (minimum log_2_) | 1339  (0.96, -0.89) | 398  (0.94, -0.91) | 149  (0.93, -0.89) | 47  (0.94, -0.95) | 0 | 1933  (0.93, -0.89) |
| FDR ≤ 10^-8^  (minimum log_2_) | 409  (1.56, -1.56) | 40  (1.79, -1.52) | 4  (1.87, ) | 0 | 0 | 453  (1.56, -1.52) |

^a^ Normalized spot intensity minus background.

^b^ Number of significant oligos in each category.

^c^ Minimum log_2_ (light/dark) value of light induced oligos.

^d^ Minimum log_2_ (light/dark) value of dark induced oligos.

L, light; D, dark.
