# Supplementary material for: Refinement of Light-Responsive Transcript Lists Using Rice Oligonucleotide Arrays: Evaluation of Gene-Redundancy
Source: PLoS One. 2008 Oct 6;3(10):e3337. doi: 10.1371/journal.pone.0003337 (PMC2556097; doi:10.1371/journal.pone.0003337)
Supplement: Table S4 — Relationship between FDR-interval values, normalized spot intensity, and maximum log2 (light/dark)-values of NSF45K light vs. dark microarray data. (0.04 MB DOC) [file pone.0003337.s004.doc]

**Table S4. Relationship of normalized spot intensities of microarray and intervals of FDR thresholds in microarray.**

| Gene list | Number of Oligos at each Spot Intensity  (minimum log_2_ light induced, dark induced) | | | | | Sum |
| --- | --- | --- | --- | --- | --- | --- |
|  | ≥1000 ^a^ | 500 – 1000 | 280 –500 | 120 – 280 | 0 – 120 |  |
| Whole array | 4,341 ^b^ | 3,572 | 3,344 | 5,275 | 26,779 | 43,311 |
| 0.05 ≤FDR≤ 0.01 | 313 ^b^  (2.48 ^c^, -1.84 ^d^) | 302  (1.29, -1.83) | 349  (1.29, -1.25) | 662  (0.92, -0.98) | 1046  (0.76, -0.92) | 2,672  (2.48, -1.84) |
| 0.01 ≤FDR≤ 10^-4^ | 911  (3.45, -3.63) | 990  (2.81, -2.96) | 997  (1.86, -1.74) | 1,483  (1.72, -1.48) | 1,032  (1.15, -1.17) | 5,413  (3.45, -3.63) |
| 10^-4^ ≤FDR≤10^-6^ | 970  (4.82, -5.24) | 808  (2.96, -2.81) | 604  (2.62, -2.34) | 553  (1.89, -1.79) | 94  (1.32, -1.01) | 3,029  (4.82, -5.24) |
| 10^-6^ ≤FDR≤10^-8^ | 930  (5.05, -5.35) | 358  (3.37, -2.78) | 145  (2.66, -1.94) | 47  (1.94, -1.36) |  | 1,480  (5.05, -5.35) |
| 10^-8^ ≤FDR≤ 10^-12^ | 409  (6.27, -5.88) | 40 (3.39, -2.27) | 4  (2.39, -) |  |  | 453  (6.27, -5.88) |
| light-induced range ^e^ /  dark-induced range ^f^ | 0.20 – 6.27/  -0.19 – -5.88 | 0.18 – 3.39/  -0.19 – -2.96 | 0.19 – 2.66/  -0.18 – -2.34 | 0.17 – 1.94/  -0.17 – -1.79 |  | 0.17 – 6.27/  -0.17 – -5.88 |

^a^ Normalized spot intensity minus background.

^b^ Number of significant oligos in each category.

^c^ Minimum log_2_ (light/dark) value of light induced oligos.

^d^ Minimum log_2_ (light/dark) value of dark induced oligos.

^e^ The range of log_2_ (light/dark) value of all light induced oligos.

^f^ The range of log_2_ (light/dark) value of all dark induced oligos.

L, light; D, dark.
