# Supplementary material for: Refinement of Light-Responsive Transcript Lists Using Rice Oligonucleotide Arrays: Evaluation of Gene-Redundancy
Source: PLoS One. 2008 Oct 6;3(10):e3337. doi: 10.1371/journal.pone.0003337 (PMC2556097; doi:10.1371/journal.pone.0003337)
Supplement: Table S5 — Summary of the number of plant GOSlim assignments for the genes that correspond to the NSF45K oligo set. There are three gene ontology (GO) principles: cellular component, biological process, and molecular function. Forty one percent of the NSF45K oligo set have at least one GOSlim term. (0.03 MB DOC) [file pone.0003337.s005.doc]

**Table S5. Plant GOSlim annotation for the NSF45K oligo set.**

| GO category | Oligos with an assigned GOSlim term | | Number of GOSlim term assignments ^c^ |
| --- | --- | --- | --- |
|  | number ^a^ | percent ^b^ |  |
| Biological process | 12,075 | 28% | 26,169 |
| Cellular component | 7,872 | 18% | 12,203 |
| Molecular function | 15,817 | 37% | 27,919 |
| Total | 17,946 | 41% | 66,291 |

^a^ Number of oligos in the NSF45K set with a GOSlim term assignment.

^b^ Percent of oligos in the NSF45K set assigned a GOSlim term in each category. (Sum exceeds 42% because many oligos have GOSlim terms in more than one category.)

^c^ Count of the number of times all GOSlim terms were assigned to an oligo in each GO category. Most oligos are assigned to more than one GO term in each category
