# Supplementary material for: Refinement of Light-Responsive Transcript Lists Using Rice Oligonucleotide Arrays: Evaluation of Gene-Redundancy
Source: PLoS One. 2008 Oct 6;3(10):e3337. doi: 10.1371/journal.pone.0003337 (PMC2556097; doi:10.1371/journal.pone.0003337)
Supplement: Table S7 — Relationship between FDR-threshold used to determine a significant gene list and expression level based on the number of ESTs in leaves of the NSF45K light vs. dark data. The number of ESTs in five selected tissues (callus, root, panicle, seed, and leaf) were compared to the FDR values from NSF45K light vs dark data. The data in this table are displayed in Figure 4. (0.05 MB DOC) [file pone.0003337.s007.doc]

**Table S7. Relationship of expression level based on the number of ESTs in leaves and significance (FDRs) of generated data.**

|  | ESTs in leaves^a^ | | | | No EST^b^ | Sum |
| --- | --- | --- | --- | --- | --- | --- |
|  | over 10 | 3~9 | 1~2 | 0 |  |  |
| Group ^c^ | 8,273 | 6,774 | 4,481 | 5,844 | 17,939 | 43,311 |
| (R^d^) | (0.19) | (0.16) | (0.10) | (0.13) | (0.41) | (1.00) |
| 0.01 ^e^ | 4,836 | 2,772 | 1091 | 683 | 979 | 10,361 |
| (R^d^) | (0.47) | (0.27) | (0.11) | (0.07) | (0.09) | (1.00) |
| 1E-04 ^e^ | 2,870 | 1,205 | 371 | 178 | 338 | 4,962 |
| (R^d^) | (0.58) | (0.24) | (0.07) | (0.04) | (0.07) | (1.00) |
| 1E-06 ^e^ | 1,333 | 377 | 96 | 36 | 91 | 1,933 |
| (R^d^) | (0.69) | (0.20) | (0.05) | (0.02) | (0.05) | (1.00) |
| 1E-08 ^e^ | 363 | 54 | 19 | 15 | 13 | 464 |
| (R^d^) | (0.78) | (0.12) | (0.04) | (0.03) | (0.03) | (1.00) |
| 1E-10 ^e^ | 18 | 2 | 0 | 0 | 0 | 20 |
| (R^d^) | (0.90) | (0.10) | (0.00) | (0.00) | (0.00) | (1.00) |

^a^ We classified expressed genes into four groups based on the number of ESTs in leaves and all digital northern data are searchable at <http://www.ricearray.org/rice_digital_northern_search.shtml>.

^b^ means the number of genes having no ESTs in every tissues analyzed by TIGR.

^c^ means the numbers of genes in above four groups.

^d^ means relative ratios of candidate genes in selected FDRs.

^e^ means selected FDRs.
