# Supplementary material for: Refinement of Light-Responsive Transcript Lists Using Rice Oligonucleotide Arrays: Evaluation of Gene-Redundancy
Source: PLoS One. 2008 Oct 6;3(10):e3337. doi: 10.1371/journal.pone.0003337 (PMC2556097; doi:10.1371/journal.pone.0003337)
Supplement: Table S9 — Summary of rice microarray data from NCBI GEO used for this study. We used the Rice Multiplatform Microarray Search Tool (http://www.ricearray.org/matrix.search.shtml) to identify oligo identifiers (ids) for genes from the different array platforms. We generated log2 fold-change data for 887 light inducible genes selected from the NSP45K light vs dark data and compared the expression of these genes with that in 20 rice Affymetrix array data sets and 19 BGI/Yale rice array data sets. More detailed information on these data is accessible at NCBI GEO (http://www.ncbi.nlm.nih.gov/geo/) with the GEO accession numbers in this table. (0.17 MB DOC) [file pone.0003337.s009.doc]

**Table S9.** Summary of rice microarray data found in NCBI GEO used for this study.

| Platform | Experiment | Comparison | # of replicates | GEO accession # | Reference |
| --- | --- | --- | --- | --- | --- |
| Affymetrix | Azucena 1ppm vs 0 ppm Sodium arsenate | Affy_Azucena 1ppm vs 0ppm Arsenate | 3 | GSE4471 |  |
| Affymetrix | Bala 1ppm vs 0 ppm Sodium arsenate | Affy_Bala 1ppm vs 0ppm Arsenate | 3 | GSE4471 |  |
| Affymetrix | Aerobic vs Anoxic from rice coleoptiles, 4 days old | Affy_Coleoptiles_aerobic vs anoxic | 2 | GSE6908 |  |
| Affymetrix | Drought (dried for 3 h) vs control (water for 3 h), young seedling tissue | Affy_Young seedling vs Drought stress | 3 | GSE6901 | (Jain et al. 2007) |
| Affymetrix | Salt (200 mM NaCl solution for 3 h) vs control (water for 3 h), young seedling tissue | Affy_Young seedling vs Salt stress | 3 | GSE6901 | (Jain et al. 2007) |
| Affymetrix | Cold (4±1 degree C for 3 h) vs control (water for 3 h), young seedling tissue | Affy_Young seedling vs Cold stress | 3 | GSE6901 | (Jain et al. 2007) |
| Affymetrix | Root (7-day-old) vs Young seedling tissue | Affy_Young seedling vs Root | 3 | [GSE6893](http://www.ncbi.nlm.nih.gov/geo/query/acc.cgi?acc=GSE6893) | (Jain et al. 2007) |
| Affymetrix | Young seedling tissue vs Mature leaf | Affy_Young seedling vs Mature leaf | 3 | [GSE6893](http://www.ncbi.nlm.nih.gov/geo/query/acc.cgi?acc=GSE6893) | (Jain et al. 2007) |
| Affymetrix | Young seedling tissue vs Shoot apical meristem | Affy_Young seedling vs SAM | 3 | [GSE6893](http://www.ncbi.nlm.nih.gov/geo/query/acc.cgi?acc=GSE6893) | (Jain et al. 2007) |
| Affymetrix | Young seedling tissue vs P1 (0-3 cm panicle, immature panicle) | Affy_Young seedling vs P1 | 3 | [GSE6893](http://www.ncbi.nlm.nih.gov/geo/query/acc.cgi?acc=GSE6893) | (Jain et al. 2007) |
| Affymetrix | Young seedling tissue vs P2 (3-5 cm panicle) | Affy_Young seedling vs P2 | 3 | [GSE6893](http://www.ncbi.nlm.nih.gov/geo/query/acc.cgi?acc=GSE6893) | (Jain et al. 2007) |
| Affymetrix | Young seedling tissue vs P3 (5-10 cm panicle, meiotic stage) | Affy_Young seedling vs P3 | 3 | [GSE6893](http://www.ncbi.nlm.nih.gov/geo/query/acc.cgi?acc=GSE6893) | (Jain et al. 2007) |
| Affymetrix | Young seedling tissue vs P4 (10-15 cm panicle, young microspore stage) | Affy_Young seedling vs P4 | 3 | [GSE6893](http://www.ncbi.nlm.nih.gov/geo/query/acc.cgi?acc=GSE6893) | (Jain et al. 2007) |
| Affymetrix | Young seedling tissue vs P5 (15-22 cm panicle, vacuolated pollen stage) | Affy_Young seedling vs P5 | 3 | [GSE6893](http://www.ncbi.nlm.nih.gov/geo/query/acc.cgi?acc=GSE6893) | (Jain et al. 2007) |
| Affymetrix | Young seedling tissue vs P6 (22-30 cm panicle, mature pollen stage) | Affy_Young seedling vs P6 | 3 | [GSE6893](http://www.ncbi.nlm.nih.gov/geo/query/acc.cgi?acc=GSE6893) | (Jain et al. 2007) |
| Affymetrix | Young seedling tissue vs S1 (0-2 dap^a^, early globular embryo) | Affy_Young seedling vs S1 | 3 | [GSE6893](http://www.ncbi.nlm.nih.gov/geo/query/acc.cgi?acc=GSE6893) | (Jain et al. 2007) |
| Affymetrix | Young seedling tissue vs S2 (3-4 dap^a^, middle and late globular embryo) | Affy_Young seedling vs S2 | 3 | [GSE6893](http://www.ncbi.nlm.nih.gov/geo/query/acc.cgi?acc=GSE6893) | (Jain et al. 2007) |
| Affymetrix | Young seedling tissue vs S3 (5-10 dap^a^, embryo morphogenesis) | Affy_Young seedling vs S3 | 3 | [GSE6893](http://www.ncbi.nlm.nih.gov/geo/query/acc.cgi?acc=GSE6893) | (Jain et al. 2007) |
| Affymetrix | Young seedling tissue S4 (11-20 dap^a^, embryo maturation) | Affy_Young seedling vs S4 | 3 | [GSE6893](http://www.ncbi.nlm.nih.gov/geo/query/acc.cgi?acc=GSE6893) | (Jain et al. 2007) |
| Affymetrix | Young seedling tissue vs S5 (21-29 dap^a^, dormancy and desiccation tolerance) | Affy_Young seedling vs S5 | 3 | [GSE6893](http://www.ncbi.nlm.nih.gov/geo/query/acc.cgi?acc=GSE6893) | (Jain et al. 2007) |
| BGI | blue light vs dark in whole seedling | BGI_Blue light vs dark | 3 | GSE2619 | (Jiao et al. 2003) |
| BGI | far-red light vs dark in whole seedling | BGI_Far-red light vs dark | 3 | GSE2619 | (Jiao et al. 2003) |
| BGI | red light vs dark in whole seedling | BGI_Red light vs dark | 3 | GSE2619 | (Jiao et al. 2003) |
| BGI | white light vs dark in root | BGI_White_root_ vs dark | 3 | GSE2619 | (Jiao et al. 2003) |
| BGI | white light vs dark in shoot | BGI_White_shoot_ vs dark | 3 | GSE2619 | (Jiao et al. 2003) |
| BGI | white light vs dark in whole seedling | BGI_White light vs dark | 3 | GSE2619 | (Jiao et al. 2003) |
| BGI | *udt1-1* (anther defect mutant) anther vs wild type anther | BGI_udt1-1 anther vs WT anther | 3 | GSE2619 | (Jung et al. 2005) |
| BGI | wild type anther 1 in meiosis vs Palea/lemma | BGI_WT anther at meiosis vs PL | 2 | GSE2619 | (Jung et al. 2005) |
| BGI | wild type anther 2 in young microspore vs Palea/lemma | BGI_WT anther at young microspore vs PL | 2 | GSE2619 | (Jung et al. 2005) |
| BGI | wild type anther 3 in vacuolated pollen vs Palea/lemma | BGI_WT anther at vacuolated pollen vs PL | 2 | GSE2619 | (Jung et al. 2005) |
| BGI | wild type anther 4 in mature pollen vs Palea/lemma | BGI_WT anther at pollen mitosis vs PL | 2 | GSE2619 | (Jung et al. 2005) |
| BGI | Seedling vs suspension cell | BGI_Seedling vs cultured cell | 3 | GSE2691 | (Ma et al. 2005) |
| BGI | Shoot vs suspension cell | BGI_Shoot vs cultured cell | 3 | GSE2691 | (Ma et al. 2005) |
| BGI | Root vs suspension cell | BGI_Root vs cultured cell | 3 | GSE2691 | (Ma et al. 2005) |
| BGI | Panicle 1 (heading panicle) vs suspension cell | BGI_Young Panicle vs cultured cell | 3 | GSE2691 | (Ma et al. 2005) |
| BGI | Panicle 2 (filling panicle) vs suspension cell | BGI_Young Panicle vs cultured cell | 3 | GSE2691 | (Ma et al. 2005) |
| BGI | Embryo vs Callus | BGI_Mature Panicle vs cultured cell | 3 | GSE6552 | (Su et al. 2007) |
| BGI | Shoot vs Embryo | BGI_Embryo vs Callus | 3 | GSE6552 | (Su et al. 2007) |
| BGI | Root vs Embryo | BGI_Shoot vs Embryo | 3 | GSE6552 | (Su et al. 2007) |
| NSF45K | Natural light vs dark in leaf, 4 varieties, 14 day old seedling | NSF45K light_vs_dark | 8 | GSE8261 | This study |

^a^ Days after pollination.

SAM indicates shoot apical meristem; P, panicle; S, developing seed; cultured cell, suspension cultured callus cells; WT, wild type anther; and PL, palea/lemma .

References for Supplemental Table S9.

Jain, M., Nijhawan, A., Arora, R., Agarwal, P., Ray, S., Sharma, P., Kapoor, S., Tyagi, A.K., and Khurana, J.P. 2007. F-Box Proteins in Rice. Genome-Wide Analysis, Classification, Temporal and Spatial Gene Expression During Panicle and Seed Development, and Regulation by Light and Abiotic Stress. *Plant Physiol* **143:** 1467-1483.

Jiao, Y., Yang, H., Ma, L., Sun, N., Yu, H., Liu, T., Gao, Y., Gu, H., Chen, Z., Wada, M., et al. 2003. A Genome-Wide Analysis of Blue-Light Regulation of Arabidopsis Transcription Factor Gene Expression During Seedling Development. *Plant Physiol* **133:** 1480-1493.

Jung, K.H., Han, M.J., Lee, Y.S., Kim, Y.W., Hwang, I., Kim, M.J., Kim, Y.K., Nahm, B.H., and An, G. 2005. Rice Undeveloped Tapetum1 Is a Major Regulator of Early Tapetum Development. *Plant Cell* **17:** 2705-2722.

Ma, L., Chen, C., Liu, X., Jiao, Y., Su, N., Li, L., Wang, X., Cao, M., Sun, N., Zhang, X., et al. 2005. A Microarray Analysis of the Rice Transcriptome and Its Comparison to Arabidopsis. *Genome Res* **15:** 1274-1283.

Su, N., He, K., Jiao, Y., Chen, C., Zhou, J., Li, L., Bai, S., Li, X., and Deng, X.W. 2007. Distinct Reorganization of the Genome Transcription Associates with Organogenesis of Somatic Embryo, Shoots, and Roots in Rice. *Plant Mol Biol* **63:** 337-349.
