# Supplementary material for: Refinement of Light-Responsive Transcript Lists Using Rice Oligonucleotide Arrays: Evaluation of Gene-Redundancy
Source: PLoS One. 2008 Oct 6;3(10):e3337. doi: 10.1371/journal.pone.0003337 (PMC2556097; doi:10.1371/journal.pone.0003337)
Supplement: Table S12 — Error rate of NSF45K light vs dark microarray data according to analysis of hph oligos. The NSF45K array contains 456 hph oligos randomly spotted throughout the two slides of the array. They are designed to anneal to the hygromycin phosphotransferase gene, a commonly used selectable marker for Agrobacterium-mediated plant transformation. In the case in which RNA from plant tissue that lacks this gene is used for the array experiment, there should be no specific annealing to these oligos and they serve as negative controls. We used their average spot intensity to calculate the background level of hybridization. (0.03 MB DOC) [file pone.0003337.s012.doc]

**Table S12. Error rate of NSF45K Light *vs* Dark Microarray Data According to Analysis of *hph* O**ligos.

| Slide  Control | Total^a^ | | Error^b^ ≥ 2-fold | | Error^c^ ≥ 4-fold | | Total error rate (%) | |
| --- | --- | --- | --- | --- | --- | --- | --- | --- |
|  | 45K_A | 45K_B | 45K_A | 45K_B | 45K_A | 45K_B | ≥2-fold | ≥ 4-fold |
| *hph*^d^ | 240 | 216 | 3^e^ | 0 | 0 | 0 | 0.66 (3/456) | 0 |
| Empty | 192 | 2,505 | 0 | 0 | 0 | 0 | 0 | 0 |

^a^ Number of control spots included on each slide.

^b^ Number of control spots showing at least 2-fold change at FDR ≤ 10^-4^.

^c^ Number of control spots showing at least 4-fold change at FDR ≤ 10^-4^.

^d^ *hph*: oligonucleotides complemetary to the hygromycin phosphotransferase gene.

^e^ FDR values of these three spots are: 4.5 X 10^-6^, 2.0 X 10^-5^ and 9.5 X 10^-5^
