# Supplementary figures and images for: Refinement of Light-Responsive Transcript Lists Using Rice Oligonucleotide Arrays: Evaluation of Gene-Redundancy
Source: PLoS One. 2008 Oct 6;3(10):e3337. doi: 10.1371/journal.pone.0003337 (PMC2556097; doi:10.1371/journal.pone.0003337)

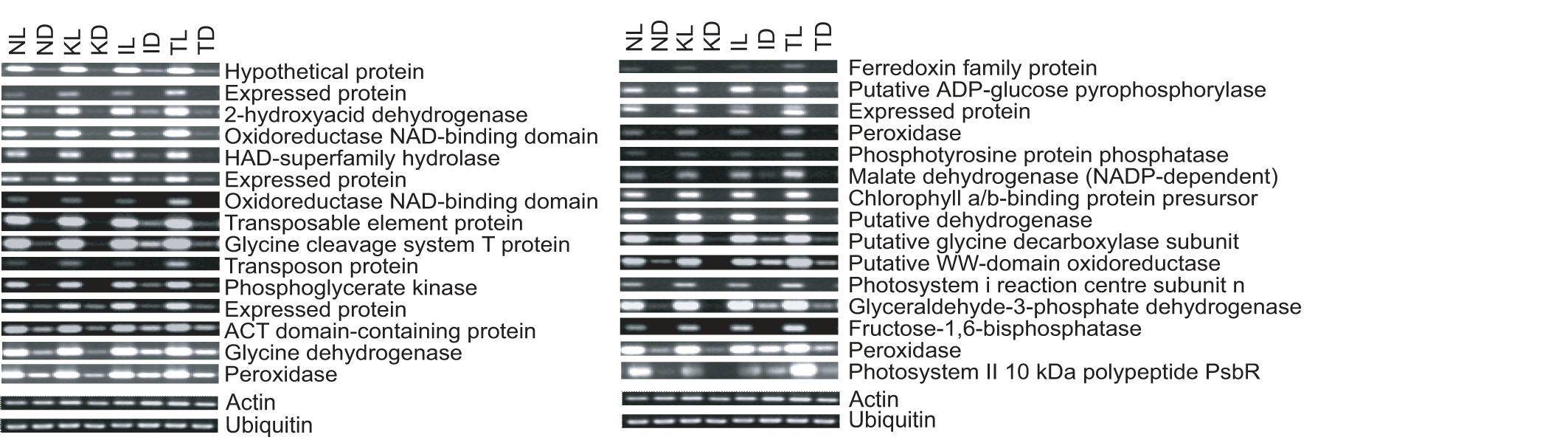

Supplement: Figure S1 — Thirty highly accumulating light-inducible genes were randomly selected for confirmation with RT-PCR with the same RNA samples applied to the NSF45K array. All tested genes consistently showed higher accumulation in the light (L) compared with the dark (D) in the 4 rice varieties (N, Nipponbare; K, Kitaake; I, IR24; T, TP309), similar to the microarray data. Rice Actin1 and Ubiquitin1 RNAs were used as internal controls [58]. (0.63 MB TIF) [file pone.0003337.s013.tif]

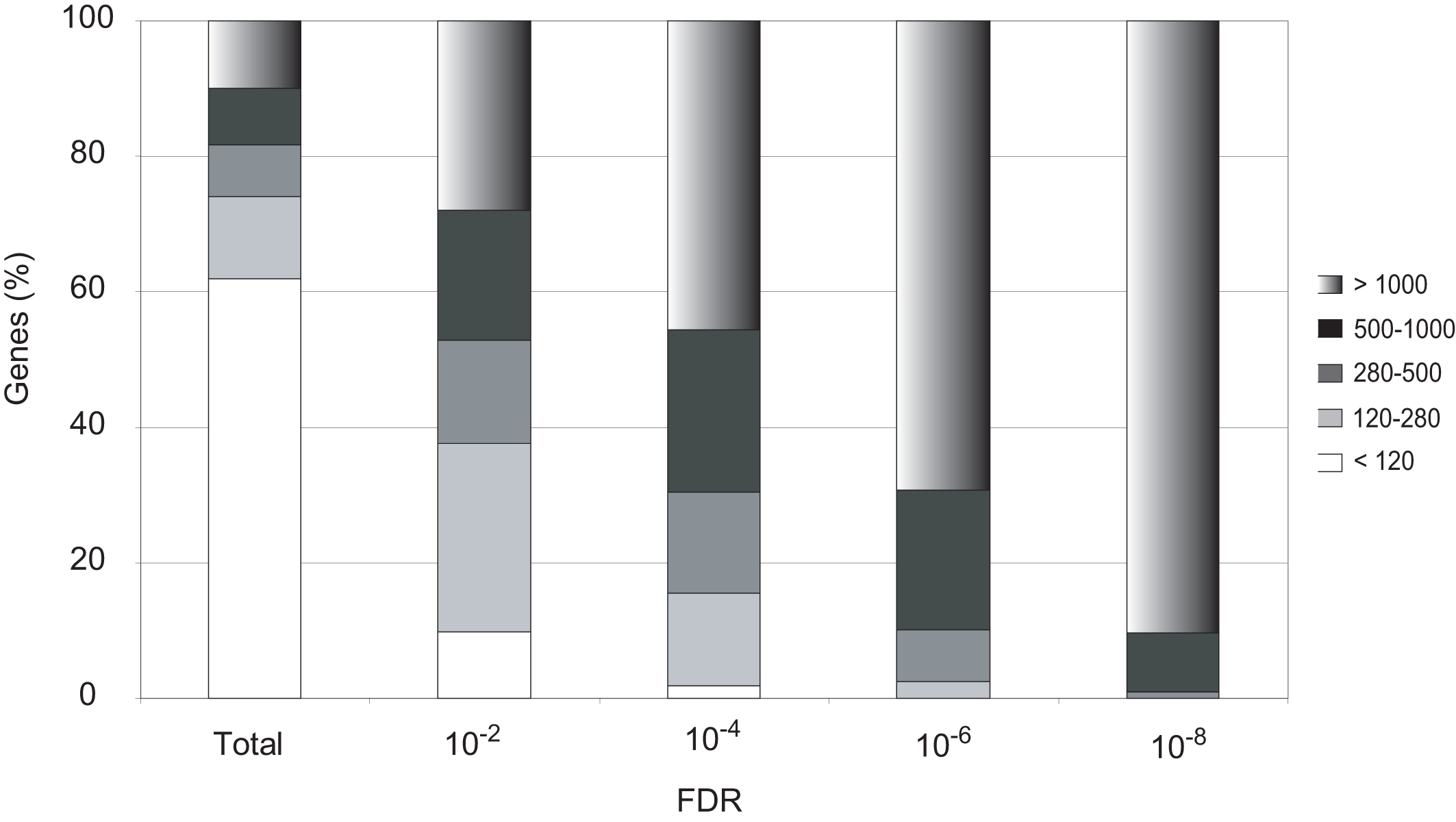

Supplement: Figure S2 — The percent of oligos represented by >1000 (grey gradient), 500–1000 (black), 280–500 (dark grey), 120–280 (light grey) or <120 normalized spot intensity (white) determined using the entire oligo set of the NSF45K array without considering FDR (total) and with genes selected from the NSF45K light vs. dark microarray data with FDR thresholds of ≤0.01, ≤10−4, ≤10−6, and ≤10−8. (1.66 MB TIF) [file pone.0003337.s014.tif]

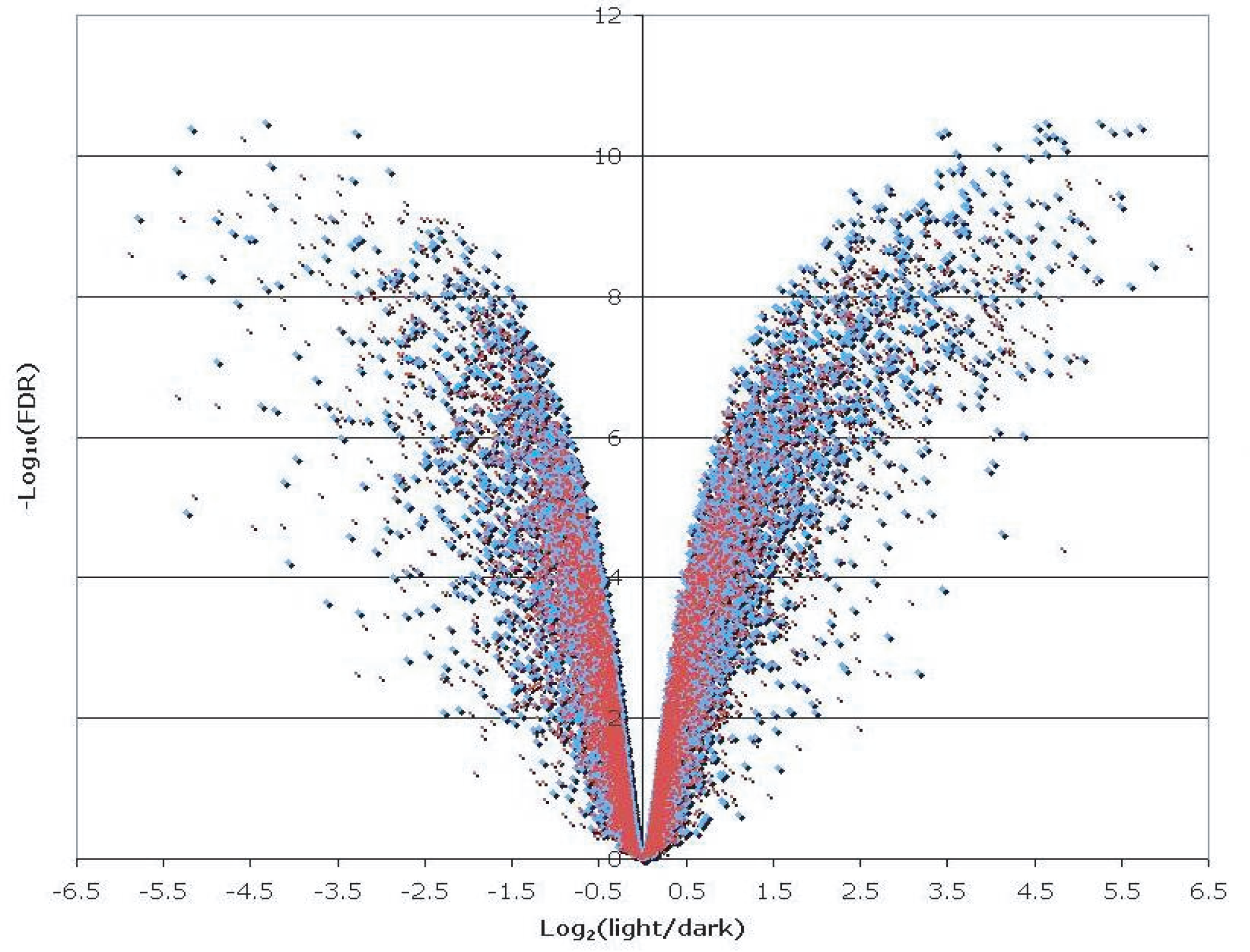

Supplement: Figure S3 — Comparison of log2 (light/dark) and (−log10) FDR-values. Blue-symbols represent data from the NSF45K slide a, and red symbols are data from the NSF45K slide b. (5.68 MB TIF) [file pone.0003337.s015.tif]

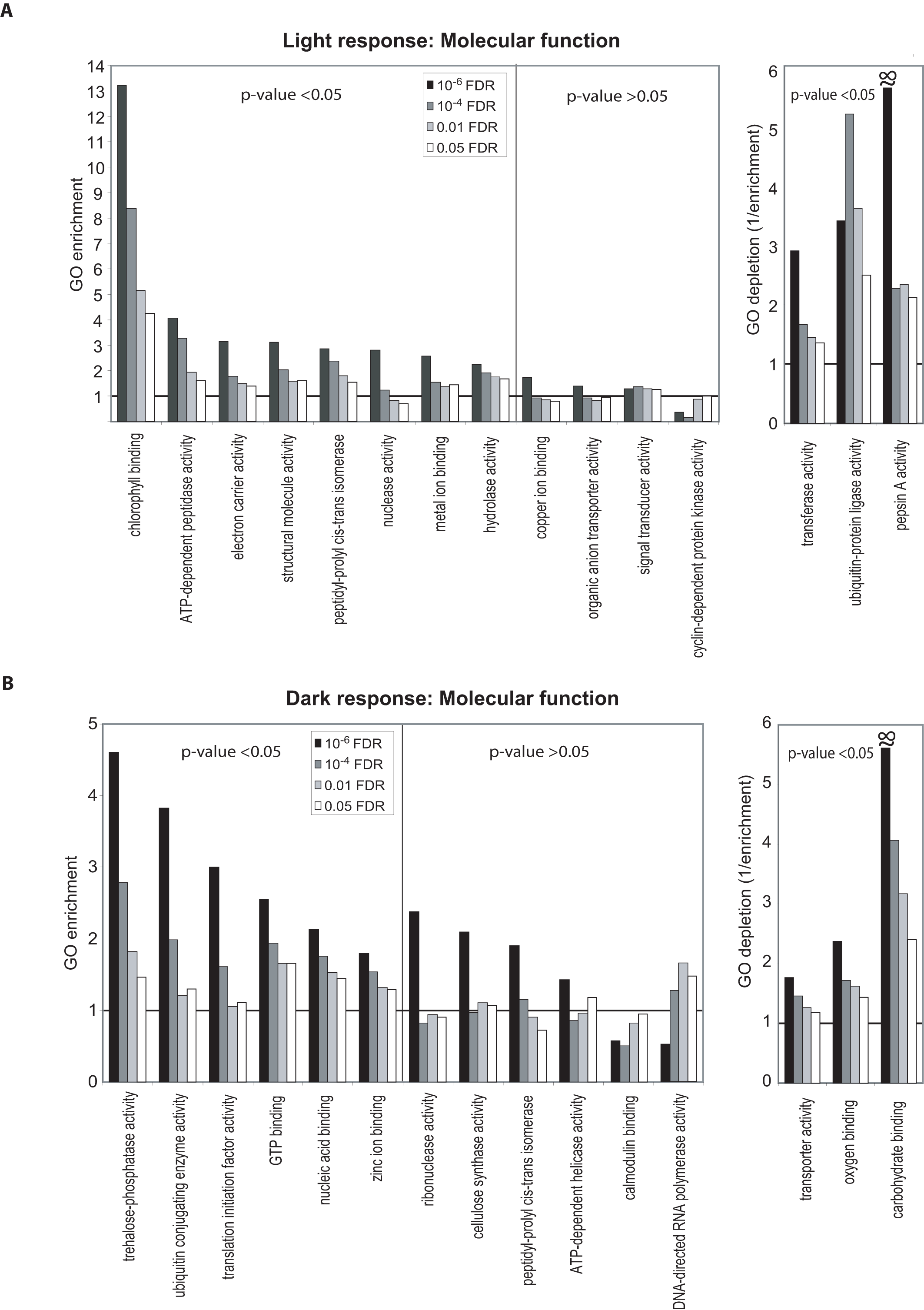

Supplement: Figure S4 — Fold-enrichment analysis of GOSlim terms in response to light or dark in the molecular function GO category. (A) GO enrichment analysis in the light. (B) GO enrichment analysis in the dark. See Figure 2 for a description of the panels. (2.06 MB TIF) [file pone.0003337.s016.tif]
